# Supplementary material for: Antiviral CD8+ T Cells Restricted by Human Leukocyte Antigen Class II Exist during Natural HIV Infection and Exhibit Clonal Expansion
Source: Immunity. 2016 Oct 18;45(4):917–30. doi: 10.1016/j.immuni.2016.09.015 (PMC5077698; doi:10.1016/j.immuni.2016.09.015)
Supplement: Document S1. Figures S1–S6, Tables S1 and S2, and Supplemental Experimental Procedures [file mmc1.pdf]

Immunity, Volume *45*

Supplemental Information

Antiviral CD8^{+} T Cells Restricted by Human Leukocyte Antigen Class II Exist during Natural HIV Infection and Exhibit Clonal Expansion

Srinika Ranasinghe, Pedro A. Lamothe, Damien Z. Soghoian, Samuel W. Kazer, Michael B. Cole, Alex K. Shalek, Nir Yosef, R. Brad Jones, Faith Donaghey, Chioma Nwonu, Priya Jani, Gina M. Clayton, Frances Crawford, Janice White, Alana Montoya, Karen Power, Todd M. Allen, Hendrik Streeck, Daniel E. Kaufmann, Louis J. Picker, John W. Kappler, and Bruce D. Walker

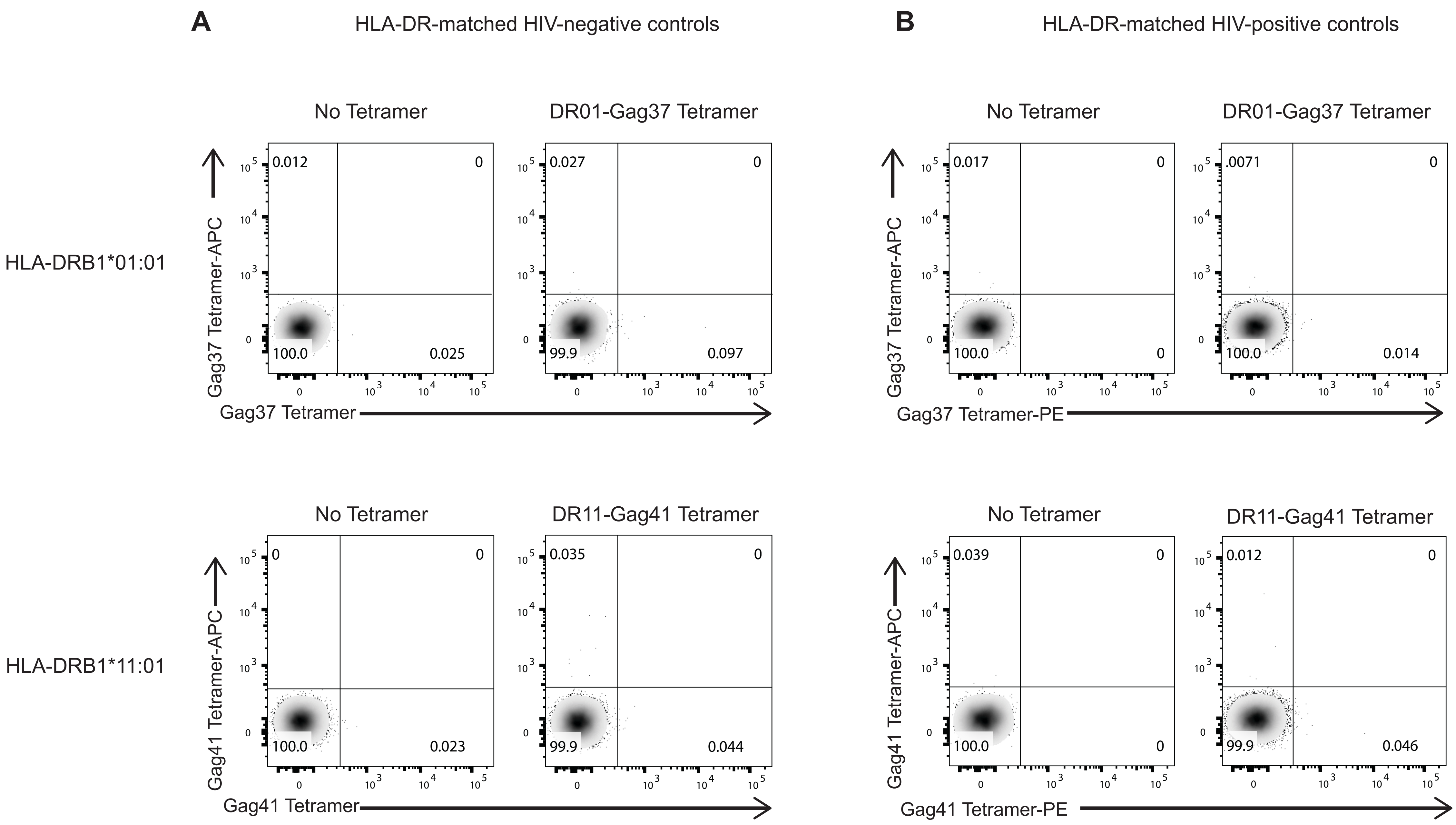

Figure S1. Related to Figure 2. HLA-DR tetramer validation in HIV-negative and HIV-positive subjects.
Representative FACS plot of HLA class II tetramer staining of fresh PBMCs in HLA-matched HIV-negative (A) and HIV-positive (B) subjects. Populations shown are gated on CD3+CD8+CD4-CD19-CD14-CD56- live lymphocyte singlets. Bulk CD8+ T cells are shown in the absence and presence of tetramer.

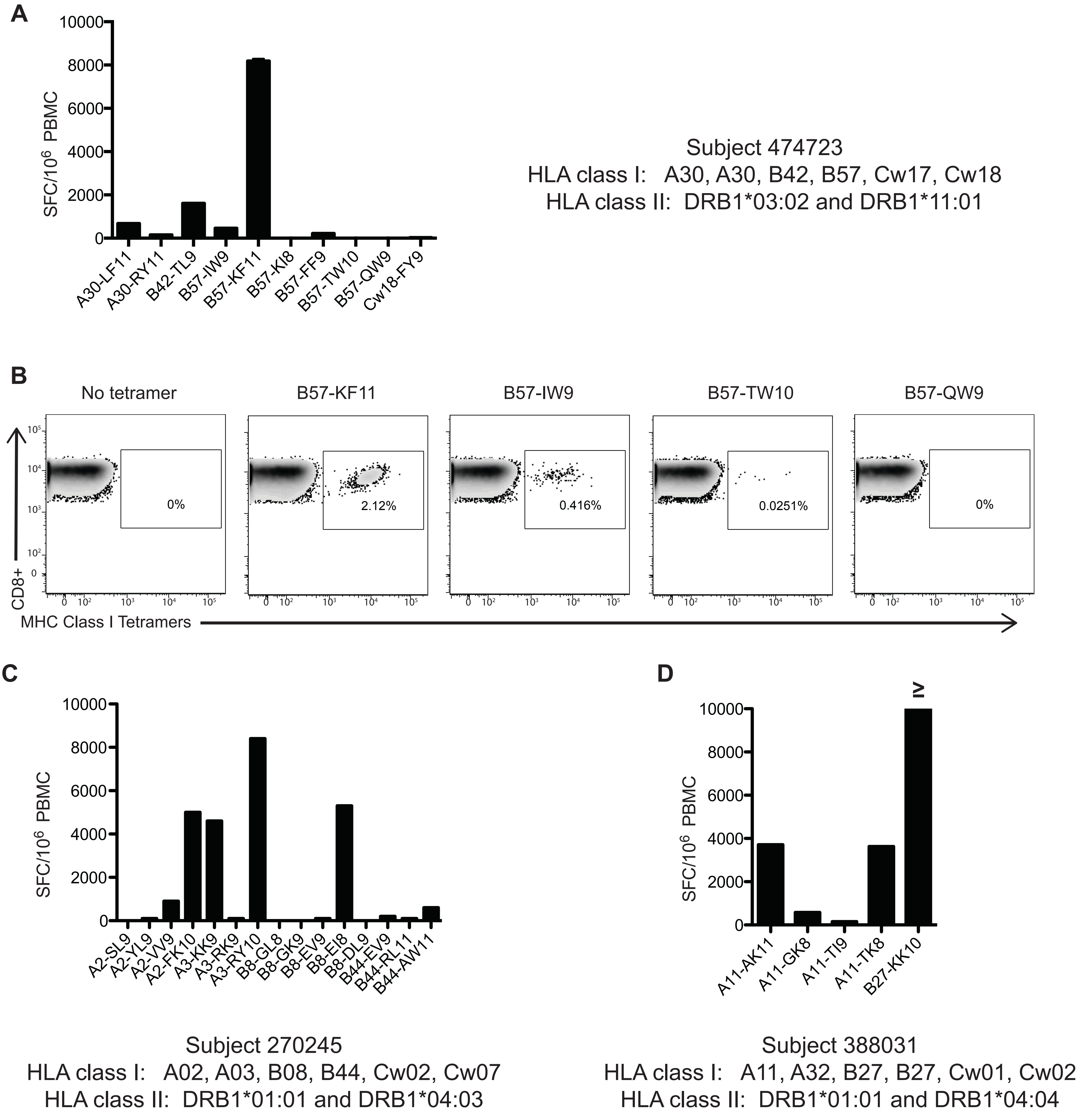

Figure S2. Related to Figure 1 and 2. Epitope-targeting of HLA class I- versus class II-restricted CD8+ T cells.
(A) Summary graph of HLA class I-restricted Gag-specific CD8+ T cells in subject 474723 as measured in a standard IFN-γ ELISPOT assay. Known optimal peptides matching the subject's HLA class I typing were added to whole PBMC. Error bars with standard deviation (SD) is shown (B) Representative FACS plots of ex-vivo tetramer positive CD8+ T cells in subject 474723 to immunodominant HLA-B*57 epitopes KF11, IW9, TW10 and QW9. (C) Summary graph of HLA class I-restricted Gag-specific CD8+ T cells in subject 270245 as measured in a standard IFN-γ ELISPOT assay. Known optimal peptides matching the subject's HLA class I were added to whole PBMC. (D) Summary graph of HLA class I-restricted Gag-specific CD8+ T cells in subject 388031 as measured in a standard IFN-γ ELISPOT assay. Known optimal peptides matching the subject's HLA class I typing were added to whole PBMC. The B*27-KK10 response was greater than the threshold of accurate detection (9999 SFU/106) as represented by "≥".

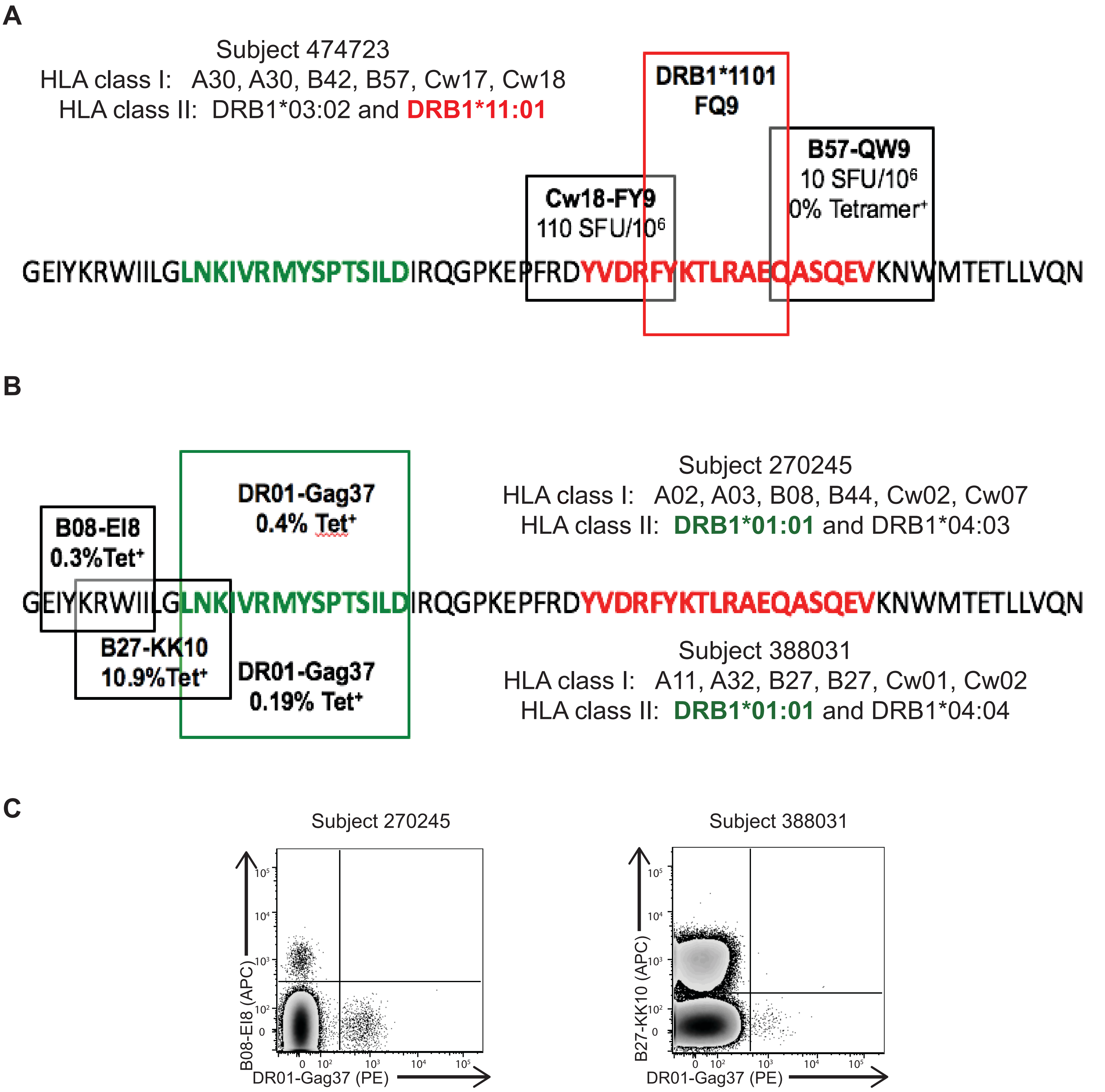

Figure S3. Related to Figure 1 and 2. Epitope-mapping of class I- versus class II-restricted CD8+ T cells. (A) Schematic of a partial Gag p24 sequence highlighting the Gag41 peptide residues in red text are shown and the predicted core peptide FA10 in a red box for subject 473723. HLA class I epitopes B*57-QW9 and Cw*18-FY9 is shown in black boxes, with values corresponding to standard IFN-γ ELISPOT and tetramer staining. (B) Schematic of a partial Gag p24 sequence highlighting the Gag37 peptide residues in green text and values corresponding to DR01-Gag37 tetramer staining for subjects 270245 and 388031 in a green box. For subject 270245, value corresponding to B*08-EI8 tetramer staining is shown in a black box. For subject 388031, value corresponding to B*27-KK10 tetramer staining is shown in a black box. For reference, the Gag41 sequence is highlighted in red text. (C) Representative FACS plot denoting percentage frequency of ex-vivo tetramer positive CD8+ T cells to DR01-Gag37 and B*08-EI8 in subject 270245, and DR01-Gag37 and B*27-KK10 in subject 388031, as determined by dual staining with HLA class I (APC) and II (PE) tetramers.

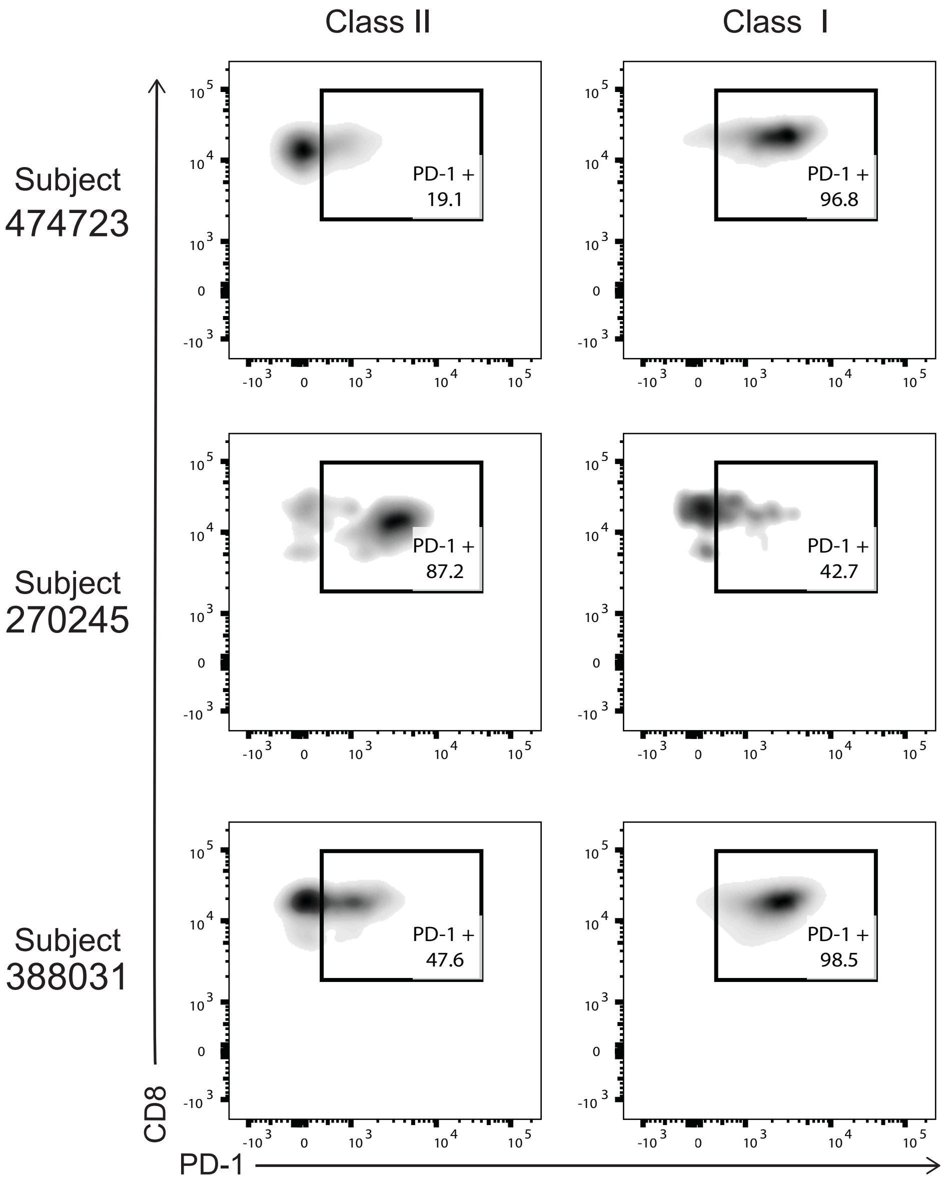

Figure S4. Related to Figure 3. PD-1 expression on class I- and class II-restricted CD8+ T cells.
Representative FACS plots of surface PD-1 expression on tetramer positive HLA class I and class II-restricted CD8+ T cells. Populations shown are gated on tetramer+ CD3+ CD8+ CD4- CD19- CD14- CD56- live lymphocyte singlets.

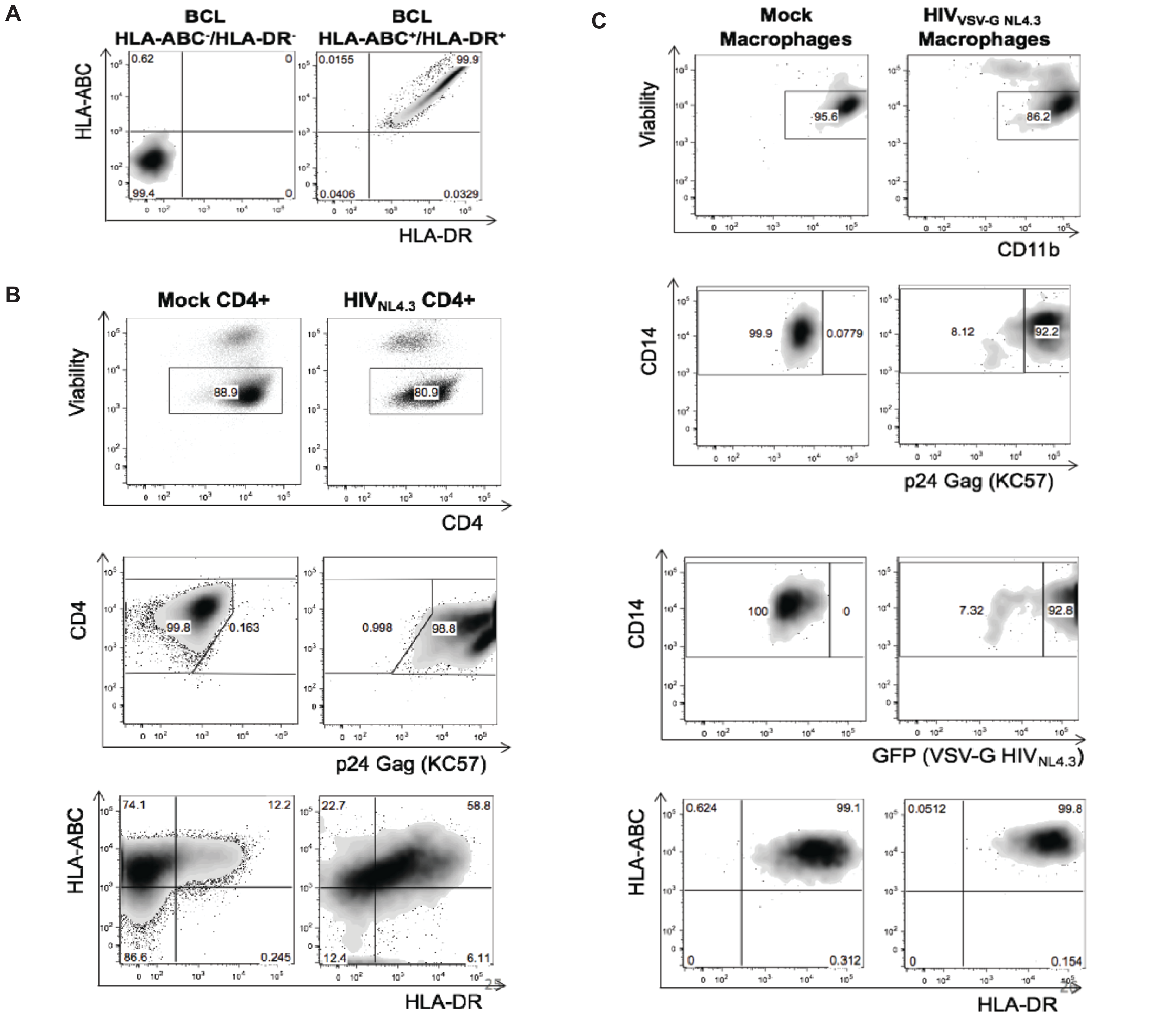

Figure S5. Related to Figure 5. HIV infection and HLA expression of Target cells.
(A) EBV-transformed B cell line, (B) CD4+ T cells, and (C) monocyte-derived macrophages from subject 474723. Representative FACS plots denoting surface HLA class I ABC and HLA-DR expression on autologous target cells using W6/32 and L432 antibodies, respectively. The proportion of intracellular Gag p24 positive CD4+ T cells was analyzed by flow cytometry 48 hrs post-infection using KC57 antibody. The CD4+ T cells were blasted with CD3+CD28+ dynabeads 3 days prior to infection by spinoculation with HIV NL4.3. The proportion of GFP positive and intracellular Gag p24 KC57 antibody positive monocyte derived macrophages (CD14+ CD11b+) was analyzed 48 hrs post-infection with VSV-G psuedotyped HIV NL4.3.

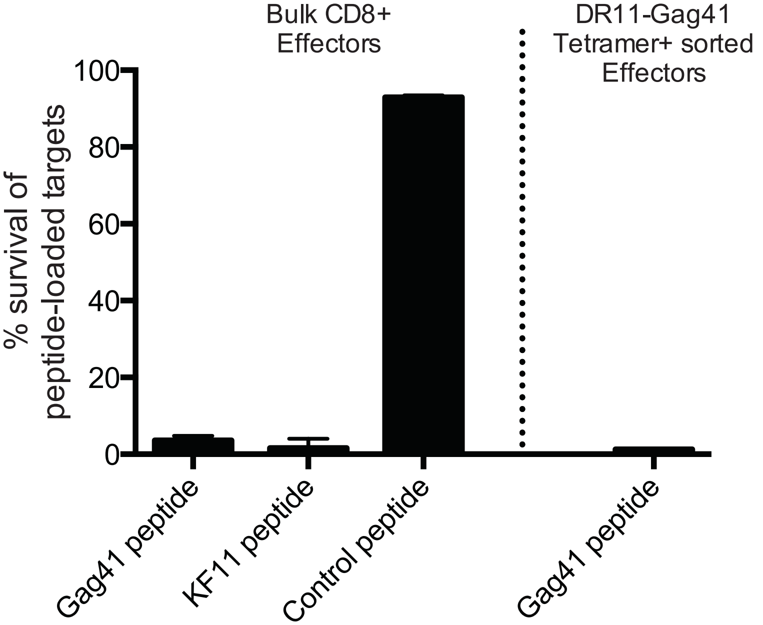

Figure S6. Related to Figure 5. Specific elimination of peptide-loaded target cells in a modified VITAL assay.

Summary graph of ex-vivo bulk CD8+ T cells and tetramer-sorted DR11-Gag41 positive cells from subject 474723 tested in a modified 36 hr VITAL assay. Ex-vivo effector cells were derived from fresh blood; processed and isolated within 12hrs. Bulk CD8+ T cells were isolated using Miltenyi CD8 MACS beads. DR11-Gag41 positive CD8+ T cells were tetramer stained and isolated by FACS. Autologous EBV-transformed BCL were used as target cells, in which half of the autologous BCL were pulsed with peptide and labeled with CFSE (Carboxyfluorescein succinimidyl ester), and the other half remained without peptide and were labeled with CTV (Cell Trace Violet), prior to co-culture with the tetramer sorted effectors in duplicate. After 36 hours, the co-culture was stained and analyzed by flow cytometry. Error bars with standard deviation shows the percentage survival of peptide-loaded cells (CFSE+).

HLA-DRB1 expression	No. of **HIV Controllers** screened by HLA-DRB1 ELISpot	No. of **HIV Controllers** screened by DR11-Gag41 tetramer	No. of **HIV Controllers** screened by DR01-Gag37 tetramer	No. of HIV Controllers with detectable DRB1-restricted CD8+ T cells
DRB1*01:01	25	-	25*	2
DRB1*04:01	18	-	-	0
DRB1*07:01	27	-	-	0
DRB1*11:01	31	68#	-	1
TOTAL	**101**	**68**	**25**	**3**

HLA-DRB1 expression	No. of **HIV Progressors** screened by HLA-DRB1 ELISpot	No. of **HIV Progressors** screened by DR11-Gag41 tetramer	No. of **HIV Progressors** screened by DR01-Gag37 tetramer	No. of HIV Progressors with detectable DRB1-restricted CD8+ T cells
DRB1*01:01	4	-	4*	0
DRB1*03:01	3	-	-	0
DRB1*07:01	7	-	-	0
DRB1*11:01	8	8#	-	0
DRB1*13:01	3	-	-	0
DRB1*15:01	3	-	-	0
TOTAL	**28**	**8**	**4**	**0**

Table S1. Related to Figure 1 and 2. Cohort of HIV-infected individuals screened for $CD8^+$ T-cell responses restricted by HLA-DR. A total of 129 HIV-infected individuals were screened for HLA-DR-restricted $CD8^+$ T-cell responses by IFN-γ Elispot and by flow cytometry using HLA class II tetramers, as depicted in the table. #DRB1*11 subjects screened by tetramer included all DR11 subjects previously screened by HLA-DRB1 Elispot. *DRB1*01 subjects screened by tetramer included all DR01 subjects previously screened by HLA-DRB1 Elispot

Ragon Identifier	474723	270245	388031
HIV status#	Viremic Controller	Viremic Controller	Elite Controller
Viral Load (RNA copies/ml) #	136	20	<75
CD4 count (cells/uL) #	1137	684	1100
HIV Diagnosis Year	1997	1982	2004
Enrolled in the Cohort	2007 until present	2005 until present	2005 until 2013
ART	1997-2003, off therapy 2003-2015, Restarted therapy Jan 2016	1987-2000, off-therapy from 2000 to present	Treatment-naive
Year of Birth	1964	1947	1966
Gender	Male	Male	Male
Race	Black or African American	White	White
HIV Risk	Blood products	MSM	MSM
Known co-infections#	HBV	TBD	TBD
HLA class I alleles	A*30:01, A*30:02, B*42:01, B*57:03, Cw17, Cw18	A*02:01, A*03:01, B*08:1, B44*05, Cw*02:02, Cw*07:01	A*11:01, A*32:01, B*27:05, B*27:05, Cw*01:02, Cw*02:02
HLA class II alleles	DRB1*03:02, DRB1*11:01, DPB*01:01, DPB*39:01, DQB*04:02, DQB*05:01,	DRB1*01:01, DRB1*04:03	DRB1*01:01, DRB1*04:01, DPB*04:01, DPB*:04:01, DQB*03:02, DQB*05:01,

Table S2. Related to Figure 2. Clinical characteristics of study subjects with detectable HLA class II-restricted $CD8^+$ T cells. The clinical characteristics for study subjects 474723, 270245, and 388031 are depicted in the table. The former two subjects are currently enrolled, however, 388031 left the cohort in 2013 with almost no sample availability remaining. Subject 474723 started antiretroviral therapy in 2016. # Information provided for principal date of assays utilizing fresh or frozen PBMC. MSM: Men who have sex with men. HBV: Hepatitis B virus

SUPPLEMENTAL EXPERIMENTAL PROCEDURES

Subjects

All study subjects gave informed consent and IRB approval was obtained from the Massachusetts General Hospital institutional regulatory board (IRB). 101 individuals recruited in this study were 'HIV Controllers', defined as HIV infected individuals who spontaneously control HIV infection in the absence of antiretroviral therapy for greater than 1 year. These HIV controllers included 40 'Elite Controllers' with viral loads of below 50 HIV RNA copies/ml for greater than 1 year, and 61 'Viremic Controllers' with viral loads of between >50 <2,000 HIV RNA copies/ml for greater than 1 year. Additionally, 28 treatment-naive HIV progressors with viral loads of greater than 2,000 HIV RNA copies/ml were screened. All subjects were chosen based on delineation of their HLA class II DRB1 alleles (with all individuals selected upon expression of one or more common DRB1 alleles spanning *01:01, *03:01, *04:01, *07:01, *11:01, *13:01 and *15:01 and availability of frozen peripheral blood mononuclear cell (PBMC) samples for HLA-DR CD8 Elispots and further functional characterization (Tables S1 and S2).

Human leukocyte antigen typing.

High resolution four-digit HLA genotyping was performed by sequence-specific PCR in accordance with standard procedures. Briefly, HLA class I–encoding genes were amplified by PCR with primers spanning exons 2 and 3, and HLA class II DRB1–encoding genes were identified by PCR amplification and sequencing of exon 2. ASSIGN 3.5 software developed by Conexio Genomics was used to interpret the sequencing results.

Peptide synthesis

Overlapping Peptides (OLPs) corresponding to HIV-1 clade B consensus 2001 for Gag protein were synthesized at the MGH Peptide Core Facility on an automated peptide synthesizer using F-moc technology. In addition, N- and C-terminal truncated peptides were synthesized for Gag41.

Elispot with whole PBMC

Screening for class I-restricted HIV-specific $CD8^+$ T cell responses was conducted using a standardized IFN-γ Elispot assay with whole PBMC, as previously described (Ranasinghe et al., 2012). In brief, whole PBMC was co-cultured individually with 10 μg/mL optimally defined class I epitopes (concordant with HLA class I typing for that individual). Input cell numbers were 100,000 whole PBMC per well and the plates were incubated overnight at 37 °C and 5% CO_2. Responses were regarded as positive if they had at least 3 times the mean background and ≥3 times the standard deviation of the negative control wells; positive responses also had to be at least 50 SFC/10^6 PBMCs.

Epitope fine-mapping

For fine-mapping analysis, $CD8^+$ T cells enriched from whole PBMC by Miltenyi $CD8^+$ MACS MicroBeads were isolated. The IFN-γ responses of $CD8^+$ T cell populations against serial truncations of Gag41 (YVDRFYKTLRAEQASQEV) restricted by DRB1*11:01 were tested. Each peptide was tested at 20 μM including, with serial truncations from the N and C termini, presented by the restricting HLA-DRB1–expressing L cell lines. Enriched $CD8^+$ T cells were co-cultured at 100,000 cells with 20,000 LCL on a modified 'HLA-DR CD8 Elispot'.

Generation of $CD8^+$ T cell clones

Whole PBMC were thawed and rested for 2 hours, with half of the PBMC then pulsed with the respective peptide of interest for 1 hour at 37 ºC, 5% CO2. After pulsing, the PBMC were washed to remove free peptide and then cultured in 60 wells of a 96-well round-bottom plate in RPMI 1640 medium containing 50 U/ml of recombinant IL-2. After approximately 2 weeks of culture, the whole PBMC TCL were then tested using the respective HLA class I or II tetramer to ascertain the percentage specificity of the population. Peptide-specific T cells were isolated using an IFN-γ secretion assay (Miltenyi), as per the manufacture's protocol. Isolated IFN-γ-positive T cells were cultured with irradiated allogeneic PBMC and CD3-specific antibody as a T cell proliferation stimulus for approximately 2 weeks and then limited dilution cloning was conducted, as previously described (Chen et al., 2012). Developing epitope-specific $CD8^+$ T cell clones were further tested separately by chromium release assays to their respective peptide, and by tetramer staining to confirm their $CD8^+$ T cell specificity. Cloned $CD8^+$ T cells were maintained by restimulation every 14 to 21 days with an anti-CD3 mAb and irradiated allogeneic PBMC in RPMI 1640 medium containing 50 U/ml of recombinant IL-2, as previously described (Ranasinghe et al., 2011) (Jones et al., 2012)

Generation of autologous targets cells for killing assays
EBV-transformed B cell lines. 10 million frozen PBMC were thawed and resuspended in 1mL of RMPI, 1.5mL of fetal bovine serum (FBS), and 1.5mL of unconcentrated supernatant of Epstein-Barr virus. Cyclosporine A (sigma) was added in a 1 µg/ml concentration. Cell were cultured for 6 to 8 weeks at 37 °C and 5% CO2
Activated CD4+ T cells. CD4+ T cell were isolated from frozen PBMC using CD4 Macs Beads (Miltenyi) and blasted with human T-activator CD3/CD28 dynabeads (ThermoFisher) for three days at 37 °C and 5% CO2
Monocyte-derived macrophages. CD14+ cells were isolated from frozen PBMC using CD14 enrichment EasySep (STEM Cell) and cultured for 6 days at 37 °C and 5% CO2 to differentiate into macrophages (CD14+ CD11b+)

Infection assessment of target cells in killing assays
To assess infectivity, target cells were surface stained with either anti-CD4 or anti-CD11b antibody, and intracellular stained with anti-p24 antibody, KC57-RD1 (Beckman Coulter). The VSV-G pseudotyped HIV NL4-3 was obtained from the NIH AIDS Reagent Program, Division of AIDS, NIAID, NIH: pNL4-3-deltaE-EGFP (Cat# 11100) from Drs. Haili Zhang, Yan Zhou, and Robert Siliciano.

Vital Assay
A modified VITAL assay (Hermans et al., 2004) was conducted with autologous EBV-transformed B cell lines. BCL were stained either with carboxyfluorescein diacetate succinimidyl ester (CFSE; Molecular Probes, Life Technologies, USA) or cell trace violet (CTV; Molecular Probes, Life Technologies) for 7 min at 37°C and then washed. 10 µg/mL of appropriate peptide was added separately to CFSE labeled cells, while CTV labeled cells remained without peptide, for 1 hour at 37 ºC, 5% CO_2. After several washes, 25,000 peptide$^+$ CFSE$^+$ cells were mixed with 25,000 peptide$^-$ CTV$^+$ cells to give a total of 50,000 targets per well. The target cells were then co-cultured with fresh Gag41 Tetramer-sorted CD8$^+$ T cells or bulk CD8$^+$ T cells, at a 1:1 E:T ratio. The co-cultures were incubated for 36 hours at 37 ºC, 5% CO_2. After 36 hours, the co-cultures were surface stained with anti-CD19, -CD3, -CD4, -CD8 antibodies, and a viability marker. Compensation was performed (including CFSE and CTV) and the fixed samples were analyzed on a LSRII flow cytometer (BD Biosciences). Analysis focused on the percentage of viable peptide-loaded CFSE$^+$ cells vs. CTV$^+$ cells, which lacked the cognate peptide.

Viral Sequencing
Genomic DNA was isolated from frozen PBMC as previously described (Miura et al., 2008). In brief, genomic DNA was isolated using the Qiagen DNA blood mini kit (Qiagen Inc., Valencia, CA) and HIV-1 Gag was amplified using nested reverse transcriptase PCR (RT-PCR). The primer sequences are available upon request. PCR products were prepared for sequencing on the 454 Genome Sequencer FLX Titanium (Roche) using standard protocols with modifications as previously described (Henn et al., 2012).

Single-Cell RNA-seq
Whole Transcriptome amplification (WTA). WTA of single cells in 96 well plates was performed with a modified SMART-Seq2 protocol, as described previously (Trombetta et al., 2014), with Maxima Reverse Transcriptase (Life Technologies) used in place of superscript II. WTA products were then cleaned with Agencourt XP DNA beads (DNA SPRI) and 80% ethanol (Beckman Coulter) and Illumina sequencing libraries were prepared using Nextera XT (Illumina). The 96 samples in each plate were pooled together, and cleaned with two 0.9x DNA SPRIs (Beckman Coulter). Library quality was assessed with a high sensitivity DNA chip (Agilent) and quantified with a high sensitivity dsDNA Quant Kit (Life Technologies). Samples were sequenced on an Illumina NextSeq 500 instrument using either 30bp paired-end reads or 150bp single-end reads.

Single-Cell RNA-Seq Preprocessing. RNA-seq reads were first trimmed using Trimmomatic (Bolger et al.,2014). Trimmed reads were aligned to the RefSeq hg38 genome and transcriptome (GRCh38.2) using TopHat (Trapnell et al., 2009) and Bowtie2 (Langmead and Salzberg, 2012) respectively. The resulting transcriptome alignments were processed by RSEM to estimate the abundance (TPM) of RefSeq transcripts (Li and Dewey, 2011).

Sample Filtering and Normalization. Considering only single-cell libraries in which we could reconstruct a productive TCR alignment (see below), we excluded from further analysis libraries with poor values for total number of reads (< 25000 reads), the percentage of aligned reads (< 10% aligned), or the percentage of detected transcripts (< 20% detected). All transcripts with lower than 10 TPM expression in more than 85% of samples were removed from the analysis, and TPM values were normalized using the "normalize.quantiles" function in the Bioconductor

preprocessCore package (Bolstad, 2011). After all filtering steps, 205 cells remained from the 228 cells that had productive TCR alignments with 3274 genes.

Single-Cell gene expression comparisons. Out of the 205 cells with reconstructed TCR sequences, 30 were $CD4^+$ and 175 were $CD8^+$ by Flow Cytometry gating. The expression of CD4, CD8A, and CD8B transcripts between the $CD4^+$ and $CD8^+$ cells was compared using Mann-Whitney-Wilcoxon test in R to determine the independence of CD4, CD8A, and CD8B expression in these populations.

TCR α and β chain sequencing

In order to reconstruct CDR3 sequences from single-cell RNA-sequencing data we developed TrapeS ("TCR Reconstruction Algorithm for Paired-End Single cells"), a software package for reconstruction of TCR sequences using short (~25bp) single cell paired-end RNA-sequencing. 150bp single-end reads were converted to artificial 49 bp read pairs for TrapeS analysis. TrapeS first takes standard genomic alignments as input and identifies the genomic segments that constitute the TCR by selecting the V and J segments expressed in the cell. Next, TrapeS takes the unmapped mates of the reads mapped to the genomic segments of the TCR and reconstructs the CDR3 region using an iterative dynamic programming algorithm. In each iteration the reads are aligned to the V and J segments, allowing only partial alignment to the ends of the segments so the reads "flank" toward the CDR3 region (flank the 3' end of the V segment and the 5' end of the J segment). Our method then extends the V and J regions using the sequence of the aligned reads, and repeats this step iteratively until the reconstructed regions overlap. TrapeS is available upon request.

Preparation of soluble DR11-Gag41

As previously described (Crawford et al., 2006; Crawford et al., 1998), soluble DR11-Gag41 was expressed as soluble protein in baculovirus infected insect cells with the Gag41 peptide covalently attached via a C-terminal flexible linker to the N-terminus of the DR11 beta chain. A stabilizing C terminal acid-base leucine zipper was added to the C-terminal end of the DR11 alpha and beta chains and a peptide tag for enzymatic biotinylation was added to the c-terminus of the DR11 alpha chain. After purification this tag was biotinylated enzymatically. To prepare fluorescent tetramers, phycoerythrin-coupled streptavidin (PE-SA) was incubated with an excess of the biotinylated DR11-Gag41 or a control mouse IA^b-p3K protein and the fluorescent complex separated from the excess MHCII by size exclusion chromatography.

Preparation of soluble TRAV6-TRBV2 TCR.

As previously described (Dai et al., 2008; Tynan et al., 2005), the TRAV6-TRBV2 V domains were expressed separately in bacterial vectors fused to human Cα or Cβ. The separate chains were denatured and solubilized from inclusion bodies, mixed and refolded to form a native TCR.

Surface plasmon resonance (SPR)

SPR studies were performed with a BIAcore 2000 instrument containing a SA biosensor chip. ~2000 resonances units (RU) of biotinylated either DR11-Gag41 or control HLA-DR52c bound to a nickel mimicking peptide were captured in separate flow cells. Various concentrations of the soluble TRAV6 and TRBV2 TCR were injected for 80 seconds at 15 uL/min. Binding affinity was calculated from the association and dissociation curves using BIAEvaluation 4.1 software after subtracting the fluid phase RU signal seen with the control DR52c complex.

SUPPLEMENTAL REFERENCES

Bolstad, B.M. (2011). preprocessCore: A collection of pre-processing functions. R package version 1.32.0.

Chen, H., Ndhlovu, Z.M., Liu, D., Porter, L.C., Fang, J.W., Darko, S., Brockman, M.A., Miura, T., Brumme, Z.L., Schneidewind, A*., et al.* (2012). TCR clonotypes modulate the protective effect of HLA class I molecules in HIV-1 infection. Nat Immunol *13*, 691-700.

Crawford, F., Jordan, K.R., Stadinski, B., Wang, Y., Huseby, E., Marrack, P., Slansky, J.E., and Kappler, J.W. (2006). Use of baculovirus MHC/peptide display libraries to characterize T-cell receptor ligands. Immunol Rev *210*, 156-170.

Crawford, F., Kozono, H., White, J., Marrack, P., and Kappler, J. (1998). Detection of antigen-specific T cells with multivalent soluble class II MHC covalent peptide complexes. Immunity *8*, 675-682.

Dai, S., Huseby, E.S., Rubtsova, K., Scott-Browne, J., Crawford, F., Macdonald, W.A., Marrack, P., and Kappler, J.W. (2008). Crossreactive T Cells spotlight the germline rules for alphabeta T cell-receptor interactions with MHC molecules. Immunity *28*, 324-334.

Henn, M.R., Boutwell, C.L., Charlebois, P., Lennon, N.J., Power, K.A., Macalalad, A.R., Berlin, A.M., Malboeuf, C.M., Ryan, E.M., Gnerre, S*., et al.* (2012). Whole genome deep sequencing of HIV-1 reveals the impact of early minor variants upon immune recognition during acute infection. PLoS Pathog *8*, e1002529.

Hermans, I.F., Silk, J.D., Yang, J., Palmowski, M.J., Gileadi, U., McCarthy, C., Salio, M., Ronchese, F., and Cerundolo, V. (2004). The VITAL assay: a versatile fluorometric technique for assessing CTL- and NKT-mediated cytotoxicity against multiple targets in vitro and in vivo. J Immunol Methods *285*, 25-40.

Jones, R.B., Garrison, K.E., Mujib, S., Mihajlovic, V., Aidarus, N., Hunter, D.V., Martin, E., John, V.M., Zhan, W., Faruk, N.F*., et al.* (2012). HERV-K-specific T cells eliminate diverse HIV-1/2 and SIV primary isolates. J Clin Invest *122*, 4473-4489.

Langmead, B., and Salzberg, S.L. (2012). Fast gapped-read alignment with Bowtie 2. Nat Methods *9*, 357-359.

Li, B., and Dewey, C.N. (2011). RSEM: accurate transcript quantification from RNA-Seq data with or without a reference genome. BMC Bioinformatics *12*, 323.

Miura, T., Brockman, M.A., Brumme, C.J., Brumme, Z.L., Carlson, J.M., Pereyra, F., Trocha, A., Addo, M.M., Block, B.L., Rothchild, A.C*., et al.* (2008). Genetic characterization of human immunodeficiency virus type 1 in elite controllers: lack of gross genetic defects or common amino acid changes. J Virol *82*, 8422-8430.

Trapnell, C., Pachter, L., and Salzberg, S.L. (2009). TopHat: discovering splice junctions with RNA-Seq. Bioinformatics *25*, 1105-1111.

Tynan, F.E., Borg, N.A., Miles, J.J., Beddoe, T., El-Hassen, D., Silins, S.L., van Zuylen, W.J., Purcell, A.W., Kjer-Nielsen, L., McCluskey, J*., et al.* (2005). High resolution structures of highly bulged viral epitopes bound to major histocompatibility complex class I. Implications for T-cell receptor engagement and T-cell immunodominance. J Biol Chem *280*, 23900-23909.
