# Supplementary material for: Antiviral CD8+ T Cells Restricted by Human Leukocyte Antigen Class II Exist during Natural HIV Infection and Exhibit Clonal Expansion
Source: Immunity. 2016 Oct 18;45(4):917–30. doi: 10.1016/j.immuni.2016.09.015 (PMC5077698; doi:10.1016/j.immuni.2016.09.015)
Supplement: Document S2. Article plus Supplemental Information [file mmc2.pdf]

Article

Immunity

Antiviral CD8$^+$ T Cells Restricted by Human Leukocyte Antigen Class II Exist during Natural HIV Infection and Exhibit Clonal Expansion

Highlights

- CD8$^+$ T cells restricted by HLA-DRB1 exist in a small number of HIV-infected persons
- These CD8$^+$ T cells exhibit potent antiviral functions against HIV-infected cells
- TCRβ usage patterns indicate clonal expansion of class II-restricted CD8$^+$ T cells
- CD8$^+$ T cells that violate immunologic paradigms may contribute to viral control

Authors

Srinika Ranasinghe, Pedro A. Lamothe, Damien Z. Soghoian, ..., Louis J. Picker, John W. Kappler, Bruce D. Walker

Correspondence

sranasinghe@mgh.harvard.edu (S.R.), bwalker@mgh.harvard.edu (B.D.W.)

In Brief

MHC class II-restricted CD8$^+$ T cells have been reported in *Cd4*-deficient mice and an SIV vaccine trial. Ranasinghe et al. demonstrate that HLA class II-restricted CD8$^+$ T cells also exist in a small percentage of HIV-infected individuals, show potent antiviral functions against HIV-infected cells, and exhibit atypical TCR usage patterns.

CrossMark

Ranasinghe et al., 2016, Immunity *45*, 917–930
October 18, 2016 © 2016 The Author(s). Published by Elsevier Inc.
http://dx.doi.org/10.1016/j.immuni.2016.09.015

CellPress

Immunity
Article

Antiviral CD8+ T Cells Restricted by Human Leukocyte Antigen Class II Exist during Natural HIV Infection and Exhibit Clonal Expansion

Srinika Ranasinghe,[1,13,14,*] Pedro A. Lamothe,[1,2,3,13] Damien Z. Soghoian,[1] Samuel W. Kazer,[1,4,5] Michael B. Cole,[6] Alex K. Shalek,[1,4,5] Nir Yosef,[1,6] R. Brad Jones,[1,7] Faith Donaghey,[1] Chioma Nwonu,[1] Priya Jani,[1] Gina M. Clayton,[3,8] Frances Crawford,[3,8] Janice White,[8] Alana Montoya,[8] Karen Power,[1] Todd M. Allen,[1] Hendrik Streeck,[9,10] Daniel E. Kaufmann,[1,11] Louis J. Picker,[12] John W. Kappler,[3,8] and Bruce D. Walker[1,2,3,4,5,*]
[1]Ragon Institute of MGH, MIT and Harvard, Cambridge, MA 01239, USA
[2]Harvard T.H. Chan School of Public Health, Harvard University, Boston, MA 02115, USA
[3]Howard Hughes Medical Institute, Chevy Chase, MD 20815, USA
[4]Institute for Medical Engineering & Science, Massachusetts Institute of Technology, Cambridge, MA 01239, USA
[5]Broad Institute, Cambridge, MA 01239, USA
[6]Department of Physics, University of California, Berkeley, CA 94720, USA
[7]George Washington University, Washington, DC 20052, USA
[8]Department of Biomedical Research, National Jewish Health, Denver, CO 80206, USA
[9]Institute for HIV Research, University Hospital, University Duisburg-Essen, Essen 45147, Germany
[10]U.S. Military HIV Research Program, Henry M. Jackson Foundation, Rockville, MD 20910, USA
[11]Centre de Recherche du Centre hospitalier de l'Université de Montréal, Montreal, QC H2X 3J4, Canada
[12]Vaccine and Gene Therapy Institute and Oregon National Primate Research Center, Oregon Health & Science University, Beaverton, OR 97006, USA
[13]Co-first author
[14]Lead Contact
*Correspondence: sranasinghe@mgh.harvard.edu (S.R.), bwalker@mgh.harvard.edu (B.D.W.)
http://dx.doi.org/10.1016/j.immuni.2016.09.015

SUMMARY

CD8+ T cell recognition of virus-infected cells is characteristically restricted by major histocompatibility complex (MHC) class I, although rare examples of MHC class II restriction have been reported in *Cd4*-deficient mice and a macaque SIV vaccine trial using a recombinant cytomegalovirus vector. Here, we demonstrate the presence of human leukocyte antigen (HLA) class II-restricted CD8+ T cell responses with antiviral properties in a small subset of HIV-infected individuals. In these individuals, T cell receptor β (TCRβ) analysis revealed that class II-restricted CD8+ T cells underwent clonal expansion and mediated killing of HIV-infected cells. In one case, these cells comprised 12% of circulating CD8+ T cells, and TCRα analysis revealed two distinct co-expressed TCRα chains, with only one contributing to binding of the class II HLA-peptide complex. These data indicate that class II-restricted CD8+ T cell responses can exist in a chronic human viral infection, and may contribute to immune control.

INTRODUCTION

Antigen-specific CD8+ T cells are a major defense against invading viruses, killing infected cells harboring non-self proteins. The first step in this process involves T cell receptor (TCR) recognition of virus-derived peptides presented on the surface of infected cells by molecules of the major histocompatibility complex (MHC) or human leukocyte antigen (HLA) in humans. The dominant paradigm of T cell recognition dictates that CD8+ T cells recognize viral peptides of 8–11 amino acids in length bound to MHC class I molecules, whereas CD4+ T cells recognize peptides of 12 or more amino acids restricted by MHC class II (Neefjes et al., 2011). During thymic selection, double-positive T cells undergo a maturation process where they are selected as either CD8+ T cells or CD4+ T cells depending on TCR binding to peptide complexed with MHC class I or class II, respectively. However, in certain cases, T cells with atypical modes of MHC restriction have been reported. CD8+ T cell responses restricted by MHC class II have been described in approximately a dozen published reports over the past two decades. These responses have been observed in *Cd4*-deficient mice, where the complete absence of CD4+ T cells led to the unexpected expansion of CD8+ T cells restricted by class II (Matechak et al., 1996; Mizuochi et al., 1988; Pearce et al., 2004; Shimizu and Takeda, 1997; Suzuki et al., 1994; Tyznik et al., 2004), and in mouse models of transplantation (Hirosawa et al., 2011). In humans, reports have also described alloreactive CD8+ T cell responses that cross-recognize HLA class I and II (Heemskerk et al., 2001; Rist et al., 2009), indicating that unconventional HLA and TCR interactions may be an important feature of allorecognition.

Remarkably, virus-specific CD8+ T cells restricted by MHC class II were recently found to be the immunodominant responses detected in the context of immunization with a

This is an open access article under the CC BY license (http://creativecommons.org/licenses/by/4.0/).

recombinant rhesus cytomegalovirus (RhCMV) vector in a macaque simian immunodeficiency virus (SIV) vaccine model (Hansen et al., 2013), which also induced non-classical MHC-E restricted CD8$^+$ T cell responses (Hansen et al., 2016). In this setting, the induction of CD8$^+$ T cells with unconventional MHC restriction was predominantly under the genetic control of the strain 68-1 RhCMV vector and the epitopes targeted by these responses did not overlap with traditional MHC class I-restricted responses. These studies demonstrate that unconventional CD8$^+$ T cell responses can be elicited by engineered RhCMV vaccine vectors and suggest that these responses may contribute to the control of viral replication (Ranasinghe and Walker, 2013).

The extent to which HLA class II-restricted CD8$^+$ T cell responses play a role in natural human immunity is unclear. To address this in a chronic human viral infection, we screened a large cohort of HIV-1-infected persons, including HIV controllers who spontaneously control virus in the absence of antiretroviral therapy. In these HIV controllers, we and others have shown that class I-restricted CD8$^+$ T cell responses, particularly HLA-B-restricted responses targeting epitopes within the highly conserved Gag protein, are associated with enhanced control of viral replication (Emu et al., 2008; Fellay et al., 2007; Kaslow et al., 1996; Kiepiela et al., 2004, 2007; Migueles et al., 2000; Pereyra et al., 2010). We now provide data showing induction of class II HLA-DR-restricted CD8$^+$ T cell responses in 3 of 101 HIV controllers tested. These class II-restricted CD8$^+$ T cells exhibited potent antiviral functions and were characterized by a single dominant TCRβ clonotype. In one individual, the HLA-DR-restricted CD8$^+$ T cells were immunodominant, comprising 12% of circulating CD8$^+$ T cells. These data reveal the rare presence of atypical CD8$^+$ T cells restricted by HLA-DR in a human viral infection and indicate that T cells that violate immunologic paradigms may shape human antiviral responses.

RESULTS

CD8$^+$ T Cell Responses Restricted by HLA-DRB1 Exist in Natural HIV Infection

Virus-specific CD8$^+$ T cells typically recognize infected cells through presentation of processed viral peptides on HLA class I molecules, but class II-restricted responses have been detected in some experimental systems and in the context of CMV vector immunization in a macaque SIV vaccine model (Hansen et al., 2013). In order to determine whether such responses exist in a chronic human viral infection, we screened 129 people with untreated HIV-1 infection, all of whom expressed common class II DRB1 alleles (Table S1). These included 101 untreated HIV controllers who maintained viral loads of less than 2,000 RNA copies/mL plasma, as well as 28 individuals with untreated chronic HIV infection exhibiting high viral loads.

Purified CD8$^+$ T cells isolated from the peripheral blood mononuclear cells (PBMCs) of each subject were co-cultured with mouse lymphoblastoid cell lines (LCL) stably expressing a single recombinant human DRB1 molecule (Ranasinghe et al., 2013; Southwood et al., 1998) matched to the donor, which had been pulsed with individual overlapping peptides spanning the HIV Gag protein. In initial screening, we identified an HIV controller, subject 474723, in whom a single Gag peptide presented by class II HLA DRB1*11:01 resulted in a robust interferon gamma (IFN-γ) Elispot response mediated by CD8$^+$ T cells (Figure 1A). This targeted peptide, Gag41 (YVDRFYKTLRAEQASQEV, aa 164–181), is also known to be presented by DRB1*11:01 for CD4$^+$ T cell recognition in HIV-infected persons (Ranasinghe et al., 2013). N- and C-terminal truncations indicated that presentation of the FYKTLRAEQA peptide contained within the larger peptide probably represented the core residues sufficient to elicit a response (Figure 1B), with phenylalanine or tyrosine likely to be the P1 anchor residue. Of the truncated peptides tested, the most robust response was to the 16 amino acid peptide DRFYKTLRAEQASQEV, which is expected to extend out of the open class II binding groove. Class II restriction of the Gag41-specific CD8$^+$ T cell response was verified using an anti-HLA-DR blocking antibody that inhibited IFN-γ production in a dose-dependent manner (Figure 1C).

To further confirm that this response was indeed mediated by class II-restricted CD8$^+$ T cells, and to more precisely define the magnitude of the response, we constructed class II tetramers with a truncated version of the Gag41 peptide (DRFYKTLRAEQASQEV) that elicited the strongest Elispot response. More than 12% of circulating CD8$^+$ T cells were DR11-Gag41 tetramer positive directly ex vivo (Figure 2A), which was further confirmed by dual staining with both allophycocyanin (APC)- and phycoerythrin (PE)-conjugated versions of the tetramer (Figure 2B).

Assessment of subjects by IFN-γ Elispot revealed that this was the only person in whom a class II-restricted CD8$^+$ T cell response could be detected to the Gag41 epitope, and this was further confirmed by staining PBMCs of 76 persons expressing HLA DRB1*11:01 (including the 39 initially screened by IFN-γ Elispot) with DR11-Gag41 tetramers (Table S1). However, extended IFN-γ Elispot screening and subsequent construction of class II tetramers revealed two additional subjects, 270245 and 388031, in whom class II-restricted CD8$^+$ T cell responses were detectable, both targeting a peptide designated Gag37 (LNKIVRMYSPTSILD, aa 136–151) restricted by HLA-DRB1*01:01. Although these responses were present at much lower frequencies than the DR11-Gag41-restricted response, dual APC and PE tetramer staining of the CD8$^+$ T cell populations using a DR01-Gag37 tetramer confirmed these were indeed class II restricted (Figure 2B). All class II tetramers showed minimal non-specific staining when tested on HLA-matched HIV-negative and HIV-positive subjects (Figure S1). Table S2 shows HLA genotyping and clinical characteristics of these three subjects with class II-restricted CD8$^+$ T cell responses.

We next compared the magnitude and specificity of the class II-restricted CD8$^+$ T cells with the most immunodominant class I-restricted Gag-specific CD8$^+$ T cells in each of the three subjects, using HLA tetramers (Figure 2C) and Elispot assays (Figure S2). In terms of magnitude, for subject 474723 the DR11-Gag41-restricted response was immunodominant over all class I-restricted Gag-specific responses tested, at 12% frequency compared to a class I B57-KF11$^+$ tetramer population of 2% frequency ex vivo. In terms of specificity, there was minimal overlap between HLA class I and class II epitopes to which responses were detected (Figure S3A). However, in the other two subjects, the unconventional responses were subdominant. The DR01-Gag37 epitope was adjacent to epitope B08-EI8 in subject 270245 and partially overlapped with immunodominant epitope

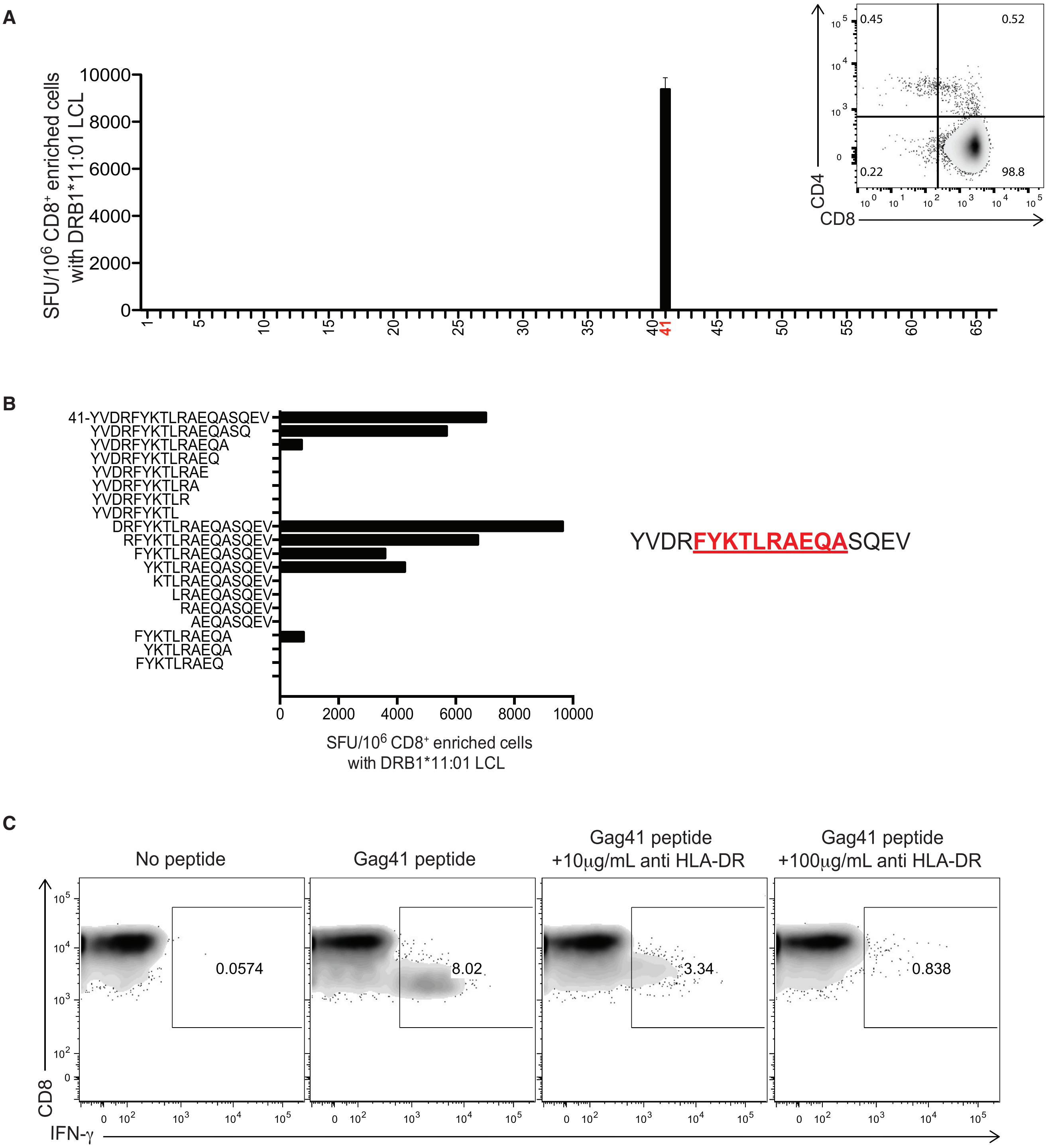

Figure 1. Elucidation of a HLA Class II DRB1-Restricted $CD8^+$ T Cell Response in HIV Controller 474723

(A) Representative IFN-γ HLA-DR Elispot using magnetically enriched $CD8^+$ T cells (FACS plot insert) co-cultured with an LCL stably transfected with DRB1*11:01 (matching the HLA type of subject) pulsed with 66 overlapping peptides (OLPs) spanning the HIV Gag protein. Error bar represents SD.

(B) Representative IFN-γ HLA-DR Elispot performed with N- and C-terminal peptide truncations presented on DRB1*11:01 LCL. Epitope FA10 within Gag41 is highlighted in red.

(C) Representative flow cytometric intracellular cytokine secretion (ICS) assay with anti-HLA-DR antibody performed on whole PBMCs co-cultured with DRB1*11:01 LCL.

See also Figures S1–S3 and Table S1.

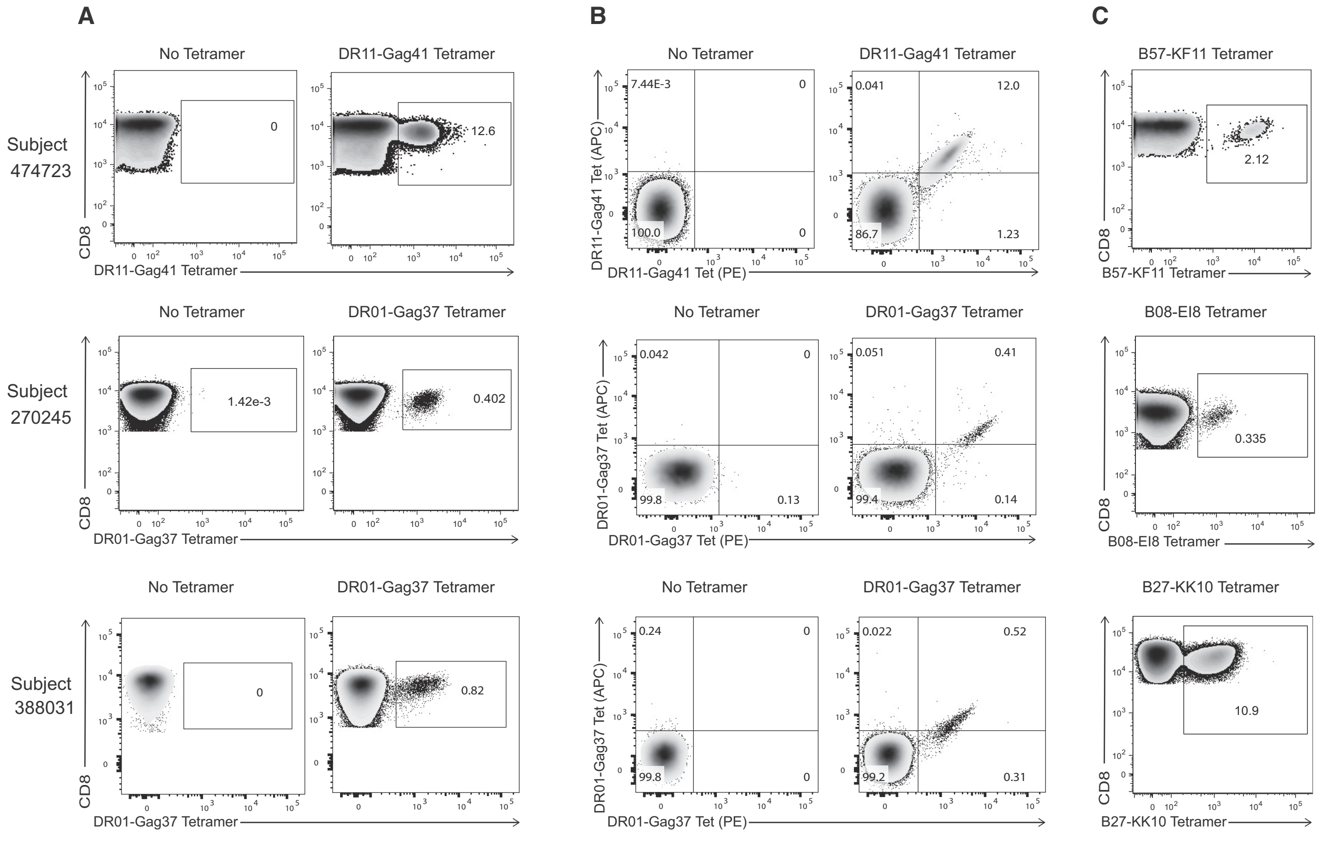

Figure 2. Detection of HLA Class II DRB1-Restricted CD8$^+$ T Cell Responses in Three HIV Controllers by Class II Tetramers

(A) Representative FACS plots of HLA class II tetramer staining using fresh PBMCs. Bulk CD8$^+$ T cells are shown in the absence and presence of DR11-Gag41 tetramer for subject 474723 and DR01-Gag37 tetramer for subjects 270245 and 388031.

(B) Representative FACS plot of dual PE and APC-conjugated class II tetramer staining.

(C) Representative FACS plots of class I tetramer staining.

All populations shown are gated on CD3$^+$CD8$^+$CD4$^-$CD19$^-$CD14$^-$CD56$^-$ live lymphocyte singlets.

See also Figures S1–S3 and Tables S1 and S2.

B27-KK10 in subject 388031, yet each population was distinct by class I and II tetramer staining (Figures S3B and S3C).

We next investigated the phenotypic characteristics of the subject-derived class II tetramer-positive CD8$^+$ T cell populations using flow cytometry (Figure 3A) and single-cell RNA sequencing (scRNA-seq) (Figure 3B). We found that both CD8α and CD8β were expressed at the protein and transcript levels. The class II-restricted CD8$\alpha\beta^+$ T cells did not co-express CD4 protein or mRNA. Next, we assessed the memory phenotype of these responses. All three subjects exhibited antigen-experienced effector memory phenotypes, although the degree of differentiation was different for each subject. In 474723, the immunodominant DR11-Gag41-positive CD8$^+$ T cells manifested an unusual differentiation for HIV-specific CD8$^+$ T cells: all tetramer-positive cells exhibited a highly differentiated effector memory (Temra) phenotype (CCR7$^-$CD45RA$^+$) (Figure 3C). In contrast, the DR01-Gag37-positive CD8$^+$ T cells, which were subdominant in vivo in both individuals, predominantly exhibited an effector memory (Tem) phenotype (CCR7$^-$CD45RA$^-$). PD-1 expression was found to be consistent with their memory phenotype (Figure S4).

Collectively, these data indicate that virus-specific CD8$\alpha\beta^+$ T cell responses restricted by HLA-DRB1 exist in the setting of natural HIV infection, and although this is a rare event, such responses can represent an immunodominant HIV-specific CD8$^+$ T cell response.

HLA Class II DRB1-Restricted CD8$^+$ T Cells Lyse Autologous HIV-Infected Targets Ex Vivo

Little is known about the function of CD8$^+$ T cells restricted by HLA class II, and particularly whether these cells have antiviral properties. Therefore, we examined the expression of granzyme B in class II-restricted CD8$^+$ T cells by intracellular staining and flow cytometry. The majority of class II tetramer$^+$ CD8$^+$ T cells from each subject was granzymeB^{hi} when compared to bulk CD8$^+$ T cells (Figure 4A). Additionally, transcriptional profiling conducted by scRNA-seq on tetramer-sorted single cells confirmed that class II-restricted CD8$^+$ T cells from all three subjects expressed perforin, granzyme B, granzyme H, MIP-1β, and RANTES (Figure 4B).

Previous studies have shown proliferation in response to epitope recognition to be associated with antiviral function (Horton et al., 2006; Riou et al., 2014), so we next compared the proliferative capacity of class II-restricted CD8$^+$ T cells with the most immunodominant class I-restricted Gag-specific CD8$^+$ T cell response in

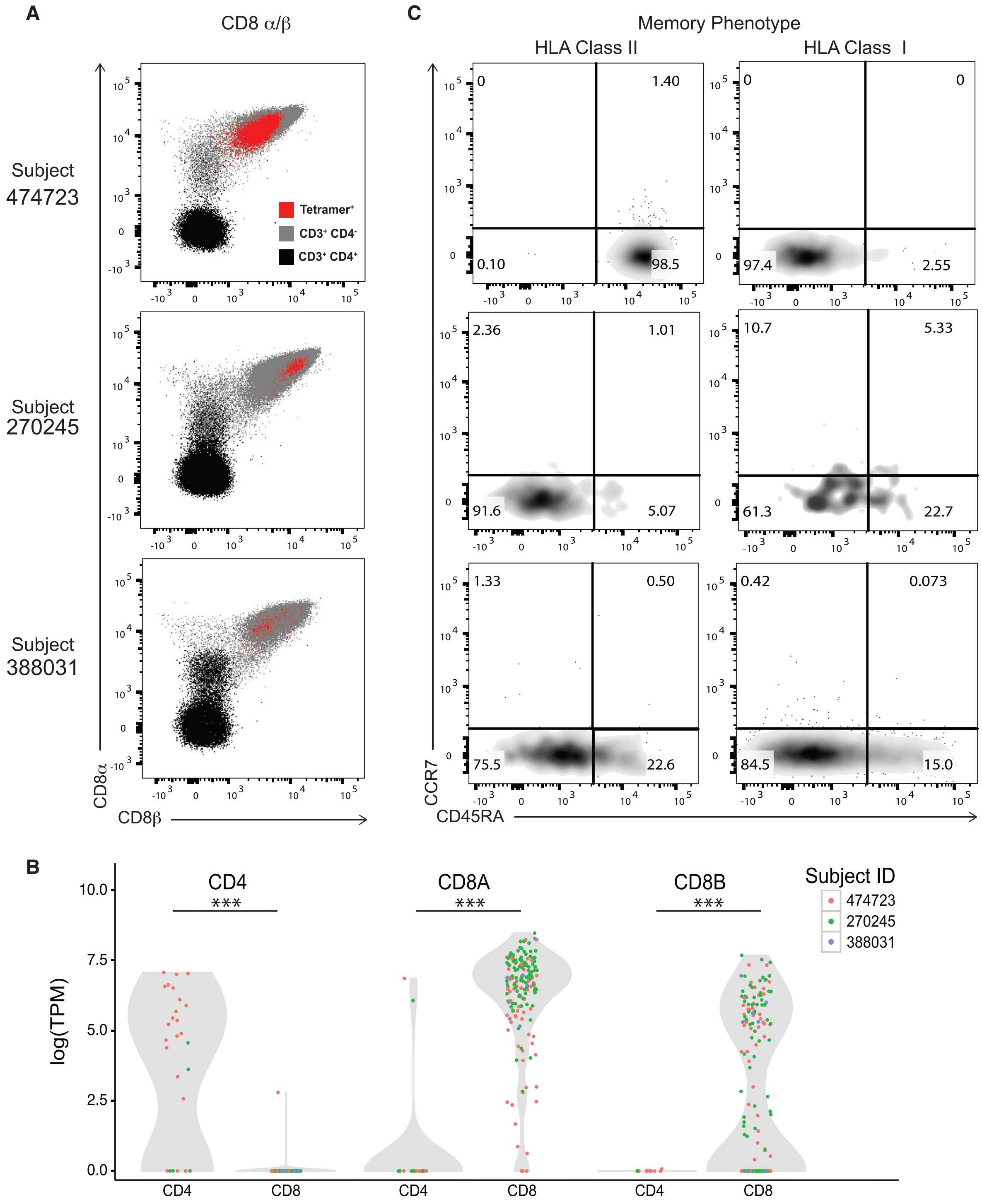

Figure 3. HLA Class II-Restricted CD8αβ T Cells in Three HIV Controllers Exhibit Heterogeneous Memory Phenotypes

(A) Representative FACS plots denoting surface expression of CD8α and CD8β. Tetramer-positive expression is shown in red. Bulk $CD3^+CD4^-$ T cells are shown in gray. $CD3^+CD4^+$ T cells are shown in black.

(B) Violin plots denoting the distribution of single-cell expression levels of CD8α (CD8A), CD8β (CD8B), and CD4 RNA transcripts in unstimulated class II tetramer-sorted $CD8^+$ and $CD4^+$ T cells estimated from single-cell RNA-seq. Abbreviation: TPM, transcripts per kilobase million. Statistical significance was determined with Mann-Whitney-Wilcoxon test (***$p < 0.001$).

(C) Representative FACS plots show memory phenotype of tetramer-positive HLA class I- and class II-restricted $CD8^+$ T cells. All populations shown are gated on $tetramer^+$ $CD3^+CD8^+CD4^-CD19^-CD14^-CD56^-$ live lymphocyte singlets.

See also Figure S4.

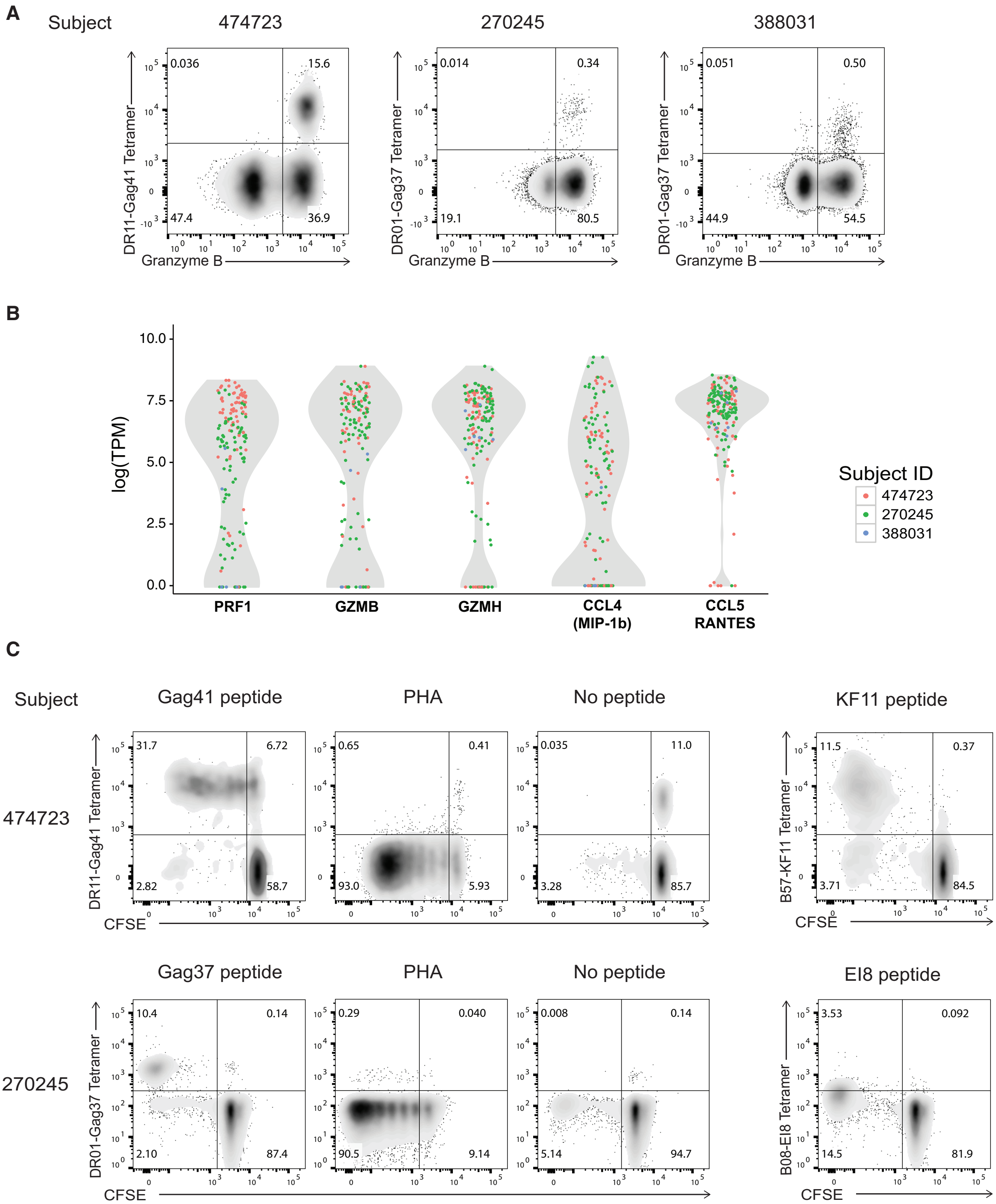

Figure 4. HLA Class II-Restricted CD8$^+$ T Cells Exhibit Cytolytic Properties and Proliferative Capacity

(A) Representative FACS plots gated on unstimulated CD8$^+$ T cells expressing intracellular granzyme B and tetramer in three HIV controllers. Tetramer populations are gated on tetramer$^+$CD3$^+$CD8$^+$CD4$^-$CD19$^-$CD14$^-$CD56$^-$ live lymphocyte singlets.

(B) Violin plots denoting the distribution of single-cell levels of Perforin, Granzyme B, Granzyme H, MIP-1b, and RANTES RNA transcripts in unstimulated class II tetramer-sorted CD8$^+$ T cells from three HIV controllers by scRNA-seq.

(C) Representative FACS plots gated on CD8$^+$ T cells expressing CFSE and tetramer at day 7 after stimulation of bulk PBMCs from HIV controllers 474723 and 270245. Abbreviations: TPM, transcripts per kilobase million; PHA, phytohaemagglutinin.

subjects 474723 and 270245, for whom sufficient samples were available. The class II tetramer-positive cells demonstrated specific proliferation upon stimulation with cognate peptide (Figure 4C). Of note, class I tetramer-positive cells from subject 270245 showed populations that were carboxyfluorescein diacetate succinimidyl ester ($CFSE^+$) positive yet tetramer negative, possibly due to TCR downregulation or non-specific proliferation induced by activation. These data demonstrate that DR11-Gag41-positive $CD8^+$ Temra cells and DR01-Gag37-positive $CD8^+$ Tem cells have substantial proliferative capacity.

We next generated class II-restricted $CD8^+$ T cell clones from subjects 474723 and 270245. These clones demonstrated specific, potent killing of autologous Epstein-Barr virus (EBV)-transformed B cells (BCL) pulsed with the cognate class II peptide. Additionally, the class II-restricted clones showed substantial cytolytic activity against autologous $CD4^+$ T cells super-infected with HIV-1 NL4.3 as well as against $CD14^+$ monocyte-derived macrophages super-infected with vesicular stomatitis virus *G*-protein (VSV-G) pseudotyped HIV-1 NL4.3-expressing GFP (Figure 5A).

Thereafter, we tested the ability of class II-restricted $CD8^+$ T cells to kill directly ex vivo and compared their antiviral efficacy with the class I-restricted $CD8^+$ T cell population (Figure 5B). BCLs, used as target cells for this assessment, displayed equivalent surface expression of HLA-ABC and HLA-DR (Figure S5). The DR11-$Gag41^+$ and B57-$KF11^+$ populations from subject 474723, as well as the DR01-$Gag37^+$ and the B08-$EI8^+$ populations from subject 270245, were tetramer sorted from fresh blood and then co-cultured with autologous BCL in a chromium release assay. The class II-restricted $CD8^+$ T cells from both subjects demonstrated direct ex vivo specific killing of peptide-pulsed BCL akin to that of the immunodominant class I-restricted $CD8^+$ T cell responses, indicating that these populations exhibit a similar killing efficiency (Figure 5B). Longer incubation of Gag41-loaded target cells with either tetramer-sorted $CD8^+$ T cells or bulk $CD8^+$ T cells from subject 474723 in a modified in Vitro Technique for Assessing Lysis (VITAL) assay resulted in greater than 98% elimination of target cells over a 36 hr incubation. The percentage of lysed target cells was similar to that achieved with bulk $CD8^+$ T cells co-cultured with KF11-loaded target cells, whereas target cells loaded with a control peptide were not eliminated (Figure S6).

We subsequently evaluated the ability of ex vivo isolated class II-restricted $CD8^+$ T cells to recognize and kill HIV-infected cells displaying naturally processed HIV antigen resulting from productive infection. Due to limited sample availability from subject 474723, we focused on autologous monocyte-derived macrophage targets that express naturally high levels of surface HLA-DR. The monocyte-derived macrophages were super-infected with a VSV-G-pseudotyped HIV-1 NL4.3 prior to co-culture with freshly isolated tetramer-sorted DR11-$Gag41^+CD8^+$ T cells. We observed that effectors lysed HIV-infected macrophages directly ex vivo, with 19% mean specific lysis compared to 4% mean specific lysis in the control ($p = 0.049$) (Figure 5C). Infection of viable macrophages, measured by GFP expression or p24 staining, was >92% (Figure S5). These data demonstrate that HIV-infected macrophages effectively process and present naturally derived HIV peptide on the cell surface for recognition by class II-restricted $CD8^+$ T cells.

As a further measure of potential $CD8^+$ T cell-mediated antiviral function, we examined the sequence of the HLA-DR-restricted epitope for evidence of immune selection pressure. More than 80% of sequences in subject 474723 showed homology to the Gag41 peptide sequence. However, almost 20% of sequences exhibited a Q308H mutation within the Gag41 epitope (Figure 5D). Although the DR11-$Gag41^+$ effectors and the bulk $CD8^+$ T cells effectively lysed BCL pulsed with wild-type peptide, they showed no detectable lysis of the Q308H peptide, consistent with this being a putative escape mutation. There was no detectable $CD8^+$ T cell response to B57-QW9 in this subject and prior sequencing of B57 controllers has not detected any Q308H mutants (Migueles et al., 2000), indicating that Gag41-specific T cells probably drive Q308H escape. These data suggest that the DR11-Gag41-restricted T cells may be capable of exerting immune selection pressure.

Taken together, our data assessing the cytolytic marker expression, epitope-specific proliferative capacity, target cell lysis, and immune selection pressure indicate that class II-restricted $CD8^+$ T cells demonstrate potent antiviral properties.

HLA Class II-Restricted $CD8^+$ T Cells Are Constituted by One Dominant TCRβ Clonotype

To further define these HLA class II-restricted $CD8^+$ T cell responses, we computationally reconstructed TCR using scRNA-seq data from single tetramer-sorted class II-restricted $CD8^+$ T cells from each of the three subjects. In each, we found that the class II-restricted $CD8^+$ T cell response was characterized by expansion of a single TCRβ clonotype (Figure 6).

The HLA class II-restricted $CD8^+$ T cell responses identified here are also immunodominant epitopes normally seen by conventional class II-restricted $CD4^+$ T cell responses (Ranasinghe et al., 2012, 2013), so we next analyzed whether both $CD4^+$ and $CD8^+$ T cells could bind the class II tetramers. The same class II tetramers that recognized $CD8^+$ T cells also stained ex vivo $CD4^+$ T cells, albeit very weakly in two of the three subjects (Figure 7A). These tetramer-positive $CD4^+$ T cells were within the expected range for an epitope-specific response (Scriba et al., 2005), yet the frequency of the class II-restricted $CD8^+$ T cell population intra-patient was dramatically larger. Together, these data reveal an immunological phenomenon in which conventional HLA class II-restricted $CD4^+$ and unconventional class II-restricted $CD8^+$ T cells can bind the same peptide-HLA complex.

We next examined whether $CD8^+$ and $CD4^+$ T cells targeting the same peptide-HLA had similar TCR rearrangements. Due to sample availability and tetramer-positive $CD4^+$ cell number constraints, we were limited to analysis of subject 474723. Because we had already identified the TCRβ V gene for the DR11-$Gag41^+$-restricted $CD8^+$ T cells, we used the class II tetramers and a TRBV2-specific fluorescent antibody to analyze both $CD8^+$ and $CD4^+$ populations (Figure 7B). Consistent with the sequencing results, we found that the class II-restricted $CD8^+$ population was comprised of 99.9% TRBV2-positive cells, and that 73.9% of the tetramer-positive $CD4^+$ response was also TRBV2 positive.

We also computationally reconstructed TCRs using scRNA-seq data from single DR11-Gag41 tetramer-sorted $CD4^+$ T cells. We identified 22 TCRβ sequences for the DR11-Gag41-restricted $CD4^+$ T cells. In contrast to the DR11-Gag41-restricted $CD8^+$, where there was only one dominant clonotype,

CellPress

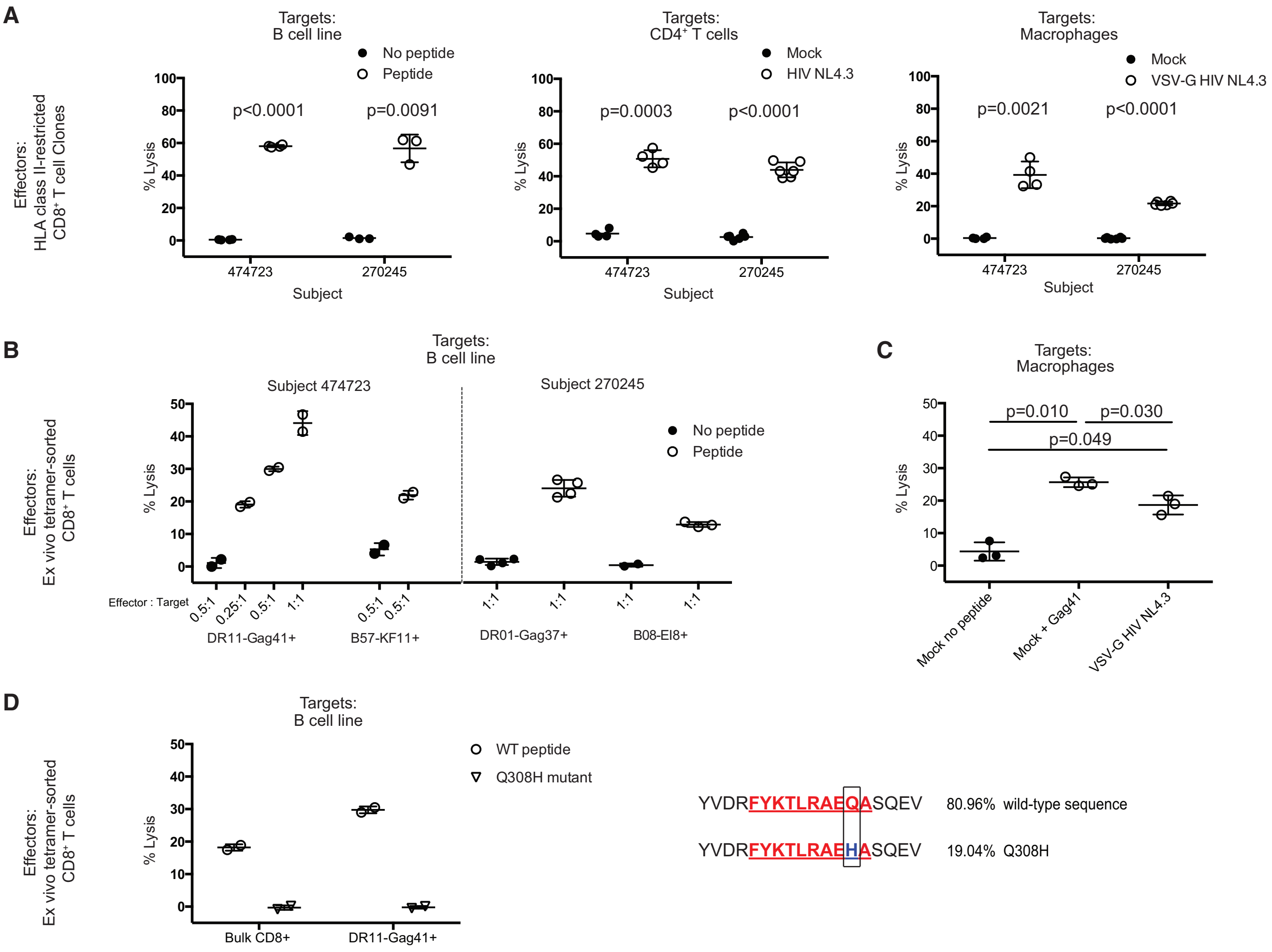

Figure 5. HLA Class II-Restricted CD8$^+$ T Cells Lyse Autologous HIV-Infected Targets Ex Vivo and Exert Putative Immune Selection Pressure In Vivo

(A) Summary data assessing specific lysis of target cells by class II-restricted CD8$^+$ T cell clones from subjects 474723 and 270245 in a standard 6 hr chromium release assay. Autologous targets included EBV-transformed BCL pulsed with peptide or no peptide, CD4$^+$ T cells infected with the HIV strain NL4.3, and monocyte-derived macrophages infected with VSV-G pseudotyped HIV NL4.3 encoding GFP, all at an effector to target cell ratio (E:T) of 1:1. Statistical significance was determined using a paired t test.

(B) Summary data assessing specific lysis of target cells by ex vivo tetramer$^+$ sorted HLA class I- and class II-restricted CD8$^+$ T cells in a standard 6 hr chromium release assay at multiple E:T ratios. Ex vivo effector cells were derived from fresh blood and tetramer sorted within 12 hr of phlebotomy. As target cells, autologous EBV-transformed BCL from subjects 474723 and 270245 were pulsed with cognate peptide or no peptide. Statistical significance was determined using a paired t test.

(C) Summary data assessing specific lysis of autologous monocyte-derived macrophages pulsed with the cognate peptide Gag41 or infected with VSV-G pseudotyped HIV NL4.3 encoding GFP by ex vivo DR11-Gag41 tetramer$^+$ sorted CD8$^+$ T cells from subject 474723 in a standard 6 hr chromium release assay at an E:T ratio of 1:1.

(D) Deep sequencing of autologous virus in subject 474723. Mutation Q308H within the Gag41 epitope is boxed. Specific lysis of EBV-transformed BCL pulsed with wild-type Gag41 peptide or the Q308H mutant peptide was tested in a standard 6 hr chromium release assay with ex vivo tetramer-sorted DR11-Gag41 tetramer$^+$ sorted CD8$^+$ T cells or bulk CD8$^+$ T cells at a 1:1 E:T ratio.

All data points for each graph represent biological replicates in a single experiment. Error bars represent SD. See also Figure S6.

we observed a more diverse response comprised of 16 different clonotypes for the CD4$^+$ T cells. Many clonotypes (13/22) used the same TRBV2 gene but had different rearrangements, highlighted in green in Figure 6. Thus in subject 474723, we found that TRBV2 is preferentially selected by both CD8$^+$ and CD4$^+$ T cell responses that target this class II HLA-peptide complex.

From characterization of the TCRα gene usages of both DR11-Gag41 CD4$^+$ and CD8$^+$ T cells using computational reconstruction from the scRNA-seq data, we obtained TCRα sequences from 71 and 17 single cells of the CD8$^+$ and CD4$^+$ populations, respectively. We found that the CD8$^+$ T cell repertoire had only one TCRβ clonotype comprised of gene segments TRBV2, TRBJ2-7, and TRBC2, but two distinct TCRα clonotypes. One was comprised of gene segments TRAV6, TRAJ39, and TRAC and the second was comprised of TRAV26-1, TRAJ16, and TRAC. However, the CD4$^+$ repertoire had ten different TCRβ

CD8⁺ T cells — TCRβ

Subject	Specificity	Clonotype Frequency	V	D	J	CDR3	C
474723	DR11-Gag41	70/70	TRBV2	TRBD2	TRBJ2-7	CASSKLASTAGEQYF	TRBC2
270245	DR01-Gag37	76/76	TRBV14	TRBD2	TRBJ2-5	CASSGGQETQYF	TRBC2
388031	DR01-Gag37	23/23	TRBV5-5	TRBD1	TRBJ1-2	CASSWDSNYGYTF	TRBC1

CD8⁺ T cells — TCRα

Subject	Specificity	Clonotype Frequency	V	J	CDR3	C
474723	DR11-Gag41	71/71	TRAV26-1	TRAJ16	CIVRPPPKGSDGQKLLF	TRAC
		67/71	TRAV6	TRAJ39	CALEGNAGNMLTF	TRAC
270245	DR01-Gag37	61/61	TRAV12-2	TRAJ4	CAVNPIGGYNKLIF	TRAC
388031	DR01-Gag37	26/26	TRAV4	TRAJ6	CLVGDGSGGSYIPTF	TRAC

CD4⁺ T cells — TCRβ

Subject	Specificity	Clonotype Frequency	V	D	J	CDR3	C
474723	DR11-Gag41	3/22	TRBV27	TRBD2	TRBJ2-7	CASSPLVPYEQYF	TRBC2
		3/22	TRBV2	TRBD2	TRBJ2-7	CASSVGASGLTEQYF	TRBC2
		1/22	TRBV2	TRBD2	TRBJ2-5	CASRPLATADTQYF	TRBC2
		1/22	TRBV2	TRBD1	TRBJ1-1	CASRGNHRGNMNTEAFF	TRBC1
		1/22	TRBV2	TRBD2	TRBJ2-7	CASSEKASGLGEQYF	TRBC2
		1/22	TRBV2	TRBD1	TRBJ1-2	CASRQGQGPFYGYTF	TRBC1
		1/22	TRBV2	TRBD2	TRBJ2-3	CASSVLTSGTDTQYF	TRBC2
		1/22	TRBV2	TRBD2	TRBJ2-2	CASRRTATSPPSFF	TRBC2
		1/22	TRBV2	TRBD2	TRBJ2-3	CASSRRTSGGTDTQYF	TRBC2
		1/22	TRBV2	TRBD2	TRBJ2-7	CASSEAASGVGEQYF	TRBC2
		1/22	TRBV2	TRBD1	TRBJ1-3	CASRATITGNTIYF	TRBC1
		1/22	TRBV2	TRBD2	TRBJ2-1	CASSRLAGGFDEQFF	TRBC2
		1/22	TRBV4-1	TRBD2	TRBJ2-7	CASSRTGGPSYEQYF	TRBC2
		1/22	TRBV5-4	TRBD2	TRBJ2-2	CASSLGDSSYTGELFF	TRBC2
		1/22	TRBV6-5	TRBD1	TRBJ1-6	CASGRGANSPLHF	TRBC1
		1/22	TRBV7-2	TRBD1	TRBJ1-2	CASSQTAQYGYTF	TRBC1
		1/22	TRBV12-3	TRBD2	TRBJ2-7	CASSPTGTGTYEQYF	TRBC2
		1/22	TRBV15	TRBD1	TRBJ1-2	CATSSGGTGRNYGYTF	TRBC1

CD4⁺ T cells — TCRα

Subject	Specificity	Clonotype Frequency	V	J	CDR3	C
474723	DR11-Gag41	8/17	TRAV24	TRAJ17	CAFKAAGNKLTF	TRAC
		1/17	TRAV24	TRAJ10	CAFVGGGNKLTF	TRAC
		1/17	TRAV17	TRAJ53	CATGGETSGSRLTF	TRAC
		1/17	TRAV17	TRAJ53	CATEEETSGSRLTF	TRAC
		1/17	TRAV17	TRAJ53	CATSGETSGSRLTF	TRAC
		1/17	TRAV14	TRAJ52	CAMREGAGGTSYGKLTF	TRAC
		1/17	TRAV20	TRAJ57	CAVQGGIQGGSEKLVF	TRAC
		1/17	TRAV3	TRAJ8	CAVRDVNTGFQKLVF	TRAC
		1/17	TRAV8-4	TRAJ32	CAVSAPYGGATNKLIF	TRAC
		1/17	TRAV8-6	TRAJ54	CAVIPIQGAQKLVF	TRAC

Figure 6. TCR Repertoires of HLA Class II-Restricted CD8⁺ T Cells Are Comprised by Monoclonal Responses

Ex vivo class II-restricted CD8⁺ and CD4⁺ T cells were tetramer sorted to be used for scRNA-seq and subsequent TCR reconstruction. TCR β and α clonotypes are shown for each identified subject, with relevant sequences highlighted in color. Relative β and α clonotype frequency was calculated as follows: number of cells with a particular clonotype / total number of cells from which any TCR Vβ or Vα gene was reconstructed, respectively. Abbreviation: CDR3, complementarity-determining region 3.

clonotypes (Figure 6). Because this analysis was performed from sorted DR11-Gag41⁺ T cells at a single cell per well for downstream RNA-seq and TCR reconstruction, we could identify that the two different TCRα chains, TRAV6-TRAJ39 and TRAV26-TRAJ16, were co-expressed within the same CD8⁺ T cell, consistent with incomplete allelic exclusion of TCRα during thymic selection (Figure 7C).

Both TRAV26 and TRAV6 sequences in the class II-restricted CD8⁺ T cells were productively rearranged. Therefore, to test for functionality, we cloned the TCR constructs and generated two TCR hybridomas that expressed either TRAV6 with TRBV2 or TRAV26 with TRBV2. Both TRAV26 and TRAV6 were able to refold properly to pair with the β, TRBV2, and be expressed on the surface of cells. However, we found that only TRAV6 (shown in red), but not TRAV26 (shown in blue), bound to the DR11-Gag41 complex when paired with TRBV2 (Figure 7D). To evaluate the functionality of the hybridomas, we measured interleukin 2 (IL-2) secretion after stimulation with the Gag41 peptide. We found that TRAV6 with TRBV2, but not TRAV26 with TRVB2, was able to produce IL-2 in a dose-dependent manner in response to peptide (Figure 7E). Both assays showed that only the TRBV2 with TRAV6 pair was functional, confirming that TRAV6 was necessary for TCR recognition of DR11-Gag41 complex. Next, we produced soluble TCR of TRAV6 with TRBV2 to assess the binding kinetics to DR11-Gag41 using surface plasmon resonance. The interaction of TRAV6 and TRBV2 with DR11-Gag41 showed an overall dissociation affinity constant (K_D) of 8.1 μM, which is typical for MHCII-peptide-specific TCRs (Figure 7F; van der Merwe and Davis, 2003). Collectively, these data indicate that these class II-restricted CD8⁺ cells express two different TCRα genes, only one of which targets HIV epitopes, possibly due to inefficient allelic exclusion during thymic T cell development, a phenomenon that is known to occur for the TCRα in ~20% of conventional T cells (Mason, 1994).

Collectively, our data from sequencing the TCRβ revealed that class II-restricted CD8⁺ T cells are monoclonal in all three subjects. Additionally, in subject 474723, a specific TRBV is preferentially selected and shared by CD8⁺ and CD4⁺ T cells targeting the same DR11-Gag41 complex. Finally, we showed that DR11-Gag41-specific CD8⁺ express two distinct TCRα chains, with only one contributing to the binding of this class II HLA-peptide complex.

DISCUSSION

CD8⁺ T cells play a critical role in control of viremia, typically through the recognition and killing of infected cells presenting pathogen-derived peptides on HLA class I molecules. Yet, whether CD8⁺ T cells restricted by HLA class II exist in natural human viral infections and exert antiviral functions is unclear. Here, we report the existence of CD8⁺ T cells that recognized HIV Gag peptides presented on HLA class II. Although these were rare events—found in 3% of the HIV controller population in this study—in one individual the class II-restricted CD8⁺ T cells were the most immunodominant CD8⁺ response detected, encompassing 12% of circulating CD8⁺ T cells. These cells exhibited high proliferative capacity and potent lysis of target cells and may have imposed selection pressure for the generation of viral escape mutants, comparable to the well-characterized antiviral efficacy of class I-restricted CD8⁺ T cells. These data illustrate that paradigm-violating HLA class II-restricted CD8⁺ T cells can be elicited in a chronic human viral infection. Moreover, our findings reveal an unexpected flexibility in CD8⁺ T cell recognition and demonstrate that the paradigm of CD8⁺ T cell restriction by HLA class I molecules is not absolute in human anti-viral immune responses.

Our analysis also revealed that class II-restricted CD8⁺ T cells demonstrated atypical patterns of TCR usage that challenge the current paradigm of T cell recognition. First, in all three subjects,

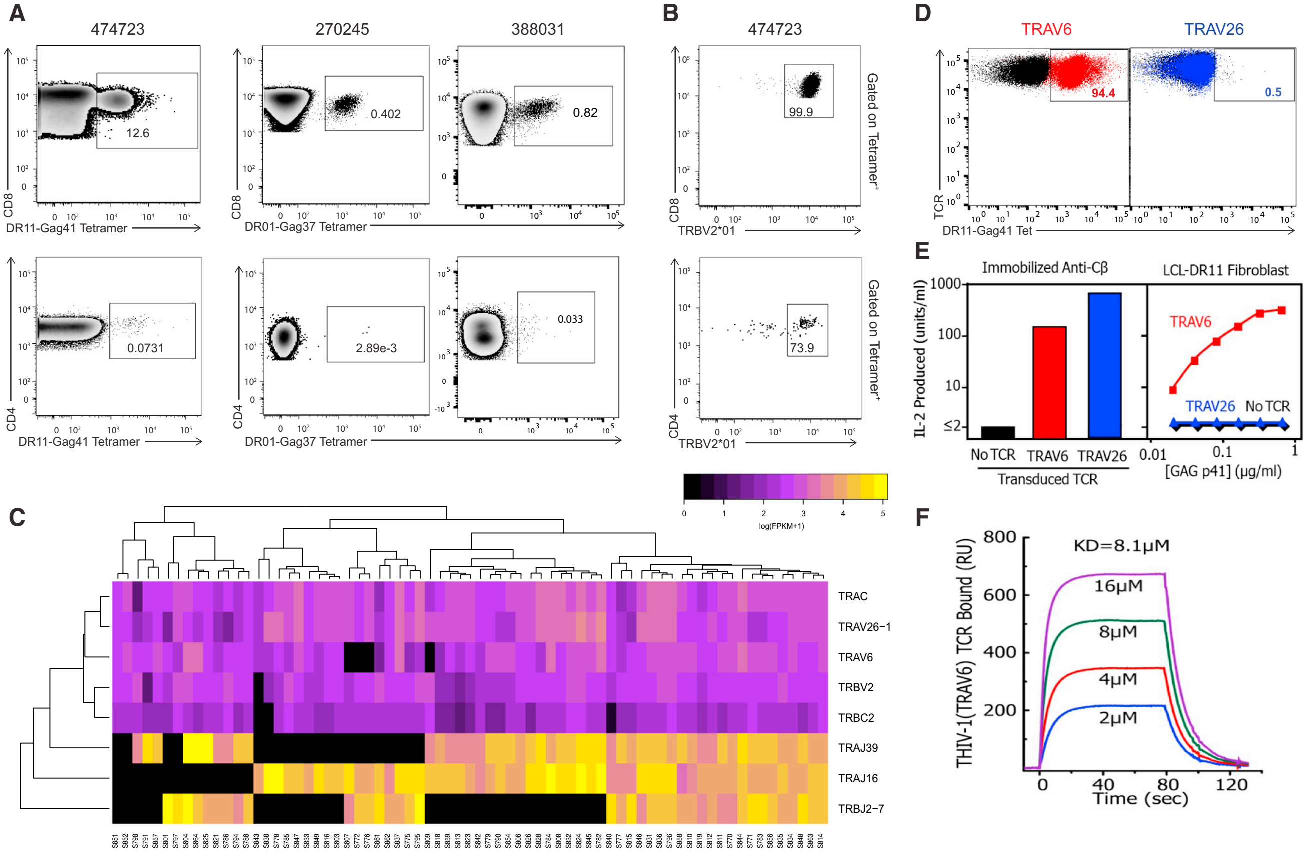

Figure 7. DR11-Gag41-Restricted CD8$^+$ T Cells Are Constituted by One Dominant TCRβ Clonotype yet Co-express Two Different TCRα Chains

(A) Representative FACS plots of CD8$^+$ T cell responses (top) and CD4$^+$ T cell responses (bottom) for each of three subjects stained with the same class II tetramers. PBMCs from subject 474723 were stained with DR11-Gag41 tetramer and those from subjects 270245 and 388031 with DR01-Gag37. All populations shown are gated on CD3$^+$CD19$^-$CD14$^-$CD56$^-$ live lymphocyte singlets.

(B) Representative FACS plot gated on CD8$^+$ and CD4$^+$ T cells from subject 474723 stained with a fluorescently conjugated TCRβ TRVB2*01-specific antibody and DR11-Gag41 tetramer. Populations shown are gated on DR11-Gag41 tetramer$^+$CD3$^+$CD19$^-$CD14$^-$CD56$^-$ live lymphocyte singlets.

(C) Heatmap showing expression of α and β TCR V, J, and C segments (rows) from individual cells (columns) from subject 474723. Ex vivo DR11-Gag41-specific CD8$^+$ T cells were tetramer sorted as single cells for scRNA-seq and subsequent TCR reconstruction.

(D) Representative FACS plot of TRAV6- and TRBV2-expressing hybridomas (left) and TRAV26- and TRBV2-expressing hybridoma (right) stained with a fluorescent HLA-DR11-Gag41 tetramer (red or blue) or no tetramer (black).

(E) Left: T cell hybridomas expressing no TCR (black) or transduced with either the TRAV6 with TRBV2 TCR (red) or the TRAV26 with TRBV2 TCR (blue) were stimulated non-antigen specifically overnight with a plate bound anti-TCR Cβ Mab to confirmed the signaling ability of the TCRs. Supernatants were assayed for secreted IL-2. Right: The same T cells were cultured overnight with an HLA-DR11-bearing LCL and various concentrations of the Gag-41 peptide. Supernatants were assayed for secreted IL-2 using the IL2-dependent cell line HT2.

(F) Biotinylated HLA DR11-Gag41 (~2,000 RU) was captured in a flow cell of a BIAcore streptavidin biosensor chip. Various concentrations of soluble TRAV6 and TRBV2 were injected for 80 s, and the affinity was calculated with BIAEvaluation 4.1 software after correction for the fluid phase RU signal.

Abbreviation: FPKM, fragments per kilobase million.

the class II-restricted CD8$^+$ T cell response was characterized by expansion of a single TCRβ clonotype, rather than the typically oligoclonal TCR repertoires observed for epitope-specific class I-restricted CD8$^+$ T cells (Almeida et al., 2007; Chen et al., 2012; Mendoza et al., 2012). Second, we observed a phenomenon in which class II-restricted CD8$^+$ T cells targeted the same HLA-peptide complex as conventional CD4$^+$ T cells. Our data indicated that, in one subject, this was associated with preferential selection of TRBV2 usage in all CD8$^+$ T cells and most CD4$^+$ T cells targeting the DR11-Gag41 complex. Although other studies have demonstrated TCRβ "public clonotypes" among virus-specific CD8$^+$ (Iglesias et al., 2011) or CD4$^+$ (Benati et al., 2016) T cells in unrelated individuals, here TCR sharing occurred between antigen-specific CD8$^+$ and CD4$^+$ T cells within an individual. Lastly, analysis of the TCRα chain revealed expression of two α chains. However, only TRAV6 in combination with TRBV2 was able to bind DR11-Gag41. We hypothesize that the second α chain (TRAV26) in combination with TRBV2 may have interacted with a class I molecule occupied by a self peptide or foreign peptide at sufficient affinity, leading to its positive selection resulting in differentiation into a CD8$^+$ single-positive T cell in the thymus with subsequent migration into the

periphery. Subsequent HIV infection may have then fortuitously selected this peripheral CD8+ T cell clone in the context of the DR11-Gag41 peptide (via its TRAV6 TCR specificity), resulting in an expanded population of memory cells with potent antiviral function. Taken together, our data imply that these unconventional CD8+ T cells exhibit distinctive TCR characteristics and suggest a mechanistic explanation as to how class II-restricted CD8+ T cells can be selected; this phenomenon may also in part explain the rarity of these responses.

Our data revealed that Gag-specific CD8+ T cells restricted by HLA-DR had a cytotoxic T lymphocyte-like phenotype and effectively killed autologous HIV-infected cells. HLA-DR-restricted CD8+ T cells may confer multiple advantages in the context of HIV infection. Unconventional restriction may allow CD8+ T cells to exert antiviral effector functions on infected macrophages and activated CD4+ T cells that typically express high levels of HLA-DR. Cytotoxic CD8+ T cells restricted by HLA-DR may also have an advantage in settings where HIV Nef-mediated class I downregulation may impair recognition (Schwartz et al., 1996). Furthermore, targeting of the Gag-37 and -41 peptides may allow HLA-DR-restricted CD8+ T cells to target a virus that has already escaped within epitopes restricted by conventional CD8+ T cells. Yet, it is difficult to assess the contribution of these unconventional CD8+ T cells to immune control. Subjects 474723 and 388031 express class I alleles B*57:03 and B*27:05, respectively, which are strongly associated with HIV-1 control (Carrington and Walker, 2012). Subject 270245 lacks these "protective" class I alleles and exhibits a DR01-Gag37-restricted CD8+ T cell response with demonstrable antiviral efficacy ex vivo. This raises the possibility that DR01-Gag37-restricted CD8+ T cell antiviral functions, in addition to CD8+ T cell responses restricted by "non-protective" class I alleles, may contribute to immune control in this individual. Further studies aimed at inducing unconventional CD8+ T cell responses in healthy humans would be required to determine their in vivo antiviral efficacy and delineate their overall contribution to control of viral replication.

The potential relevance of class II-restricted CD8+ T cell responses is underscored by results from an SIV vaccine trial. In monkeys immunized with strain 68-1 RhCMV vector and challenged with pathogenic SIV, two-thirds of the CD8+ T cell responses recognized a wide breadth of SIV Gag epitopes bound to class II molecules (Hansen et al., 2013). Induction of class II-restricted CD8+ T cells, which occurs in every immunized animal, is a consequence of the absence of two viral genes (*Rh157.5* and *Rh157.6*) in the strain 68-1 vector, as indicated by the fact that repair of these two genes reverts CD8+ T cell responses back to class I restriction (Hansen et al., 2013). The *Rh157.5* and *Rh157.4* gene products are part of a RhCMV receptor for non-fibroblasts and their absence changes the cellular tropism of the vector, making it more fibroblast-tropic, which in turn is thought to change the priming environment to favor generation of class II-restricted CD8+ T cells. As the modified vector does not change the CD8+ naive T cell repertoire, the implication of these findings is that atypical priming conditions efficiently prime pre-existing CD8+ T cells with cross-reactive TCR. Class II-restricted CD8+ T cell responses were also recently seen in 4 of 12 unvaccinated SIV-infected monkeys with controlled viremia (1 such response per "SIV contoller" monkey; 4 MHC-II-restricted responses out of a total of 180 epitope-specific responses evaluated) (Hansen et al., 2016). These data support our findings that memory Gag-specific CD8+ T cell responses restricted by class II can be elicited in natural viral infection, and as such must exist in the naive T cell repertoire of at least some humans and macaques. Thus it may be possible to induce and expand these responses in healthy uninfected subjects. However, we currently do not know whether class II-restricted CD8+ T cells responses actually contribute to viral control in vivo in either the CMV vector-induced or natural SIV/HIV infection models.

Although we showed that class II-restricted CD8+ T cells can exist in natural HIV infection, we note a number of limitations in this study. We detected only a single Gag-specific CD8+ T cell response restricted to HLA-DRB1 in each of three HIV controller individuals and in none of the HIV chronic progressors. The low number of responses detected may be due to the method of screening, a modified IFN-γ Elispot using LCL stably expressing a single recombinant HLA-DR molecule. Arguably, the reliance on IFN-γ detection may thwart detection of unconventional CD8+ T cell responses if they do not secrete this cytokine. To circumvent this limitation, we also screened HIV-infected individuals with class II tetramers, but CD8+ T cell responses were found only in the aforementioned three individuals, confirming that the modified Elispot is unlikely to have missed low-level responses. Because the macaque studies evaluated only SIV Gag-specific CD8+ T cell responses restricted by Mamu-DRB, we focused this study on HIV Gag-specific CD8+ T cell responses restricted by common HLA-DRB1 alleles. We did not test for class II-restricted CD8+ T cell responses to other HIV proteins, or to class II DRB4, DRB5, DP, or DQ. Another constraint in our study was limited sample availability and low numbers of tetramer-positive cells, so in some parts of this study, we primarily focused on the characterization of subject 474723. This subject demonstrated potent killing of target cells ex vivo, showed putative evidence of viral escape in vivo, and exhibited unique TCR features. However, given the rarity of these unconventional CD8+ T cell responses, it is not clear whether we can make generalizations between class I- and class II-restricted CD8+ T cells. Indeed, further work will be required to determine whether these unconventional responses represent a distinct subset of HIV-responsive cells or represent class I-restricted CD8+ T cells that simply happen to bear TCR that cross-react with Gag peptide presented by class II. Finally, whether these results can be extrapolated to unconventional T cells in other pathogenic infections or vaccine settings will require additional study.

In summary, these data reveal rare class II-restricted CD8+ T cell responses with potent antiviral properties and clonal expansion in the setting of a natural human viral infection, challenging current paradigms of T cell recognition and restriction. Our findings suggest greater flexibility in CD8+ T cell recognition and restriction, which is likely modulated by TCR cross-reactivity and which may be important for immunological outcomes. Thus, these data not only enhance our understanding of the basic immunology of TCR-peptide-HLA interactions, but also may be important for future T cell-based vaccine design and immunotherapeutic interventions, where induction of unconventional class II-restricted CD8+ T cells that show antiviral efficacy may be beneficial.

EXPERIMENTAL PROCEDURES

Subjects

A total of 129 HIV-infected individuals were recruited from Massachusetts General Hospital after providing informed consent. From those, 101 individuals were defined as "HIV controllers:" HIV-infected individuals who spontaneously control HIV infection in the absence of antiretroviral therapy for greater than 1 year. Additionally, 28 treatment-naive HIV progressors with plasma viral loads of greater than 2,000 HIV RNA copies/mL were utilized (see also Tables S1 and S2 and Supplemental Experimental Procedures).

HLA-DR CD8$^+$ Elispot

Screening for class II-restricted HIV-specific CD8$^+$ T cell responses and epitope fine mapping was conducted by enzyme-linked immunospot (Elispot) assay, using CD8$^+$ T cells enriched by CD8 MACS MicroBeads selection (Miltenyi). Antigen-presenting cells expressing the HLA-DRB1 of the subject consisted of mouse lymphoblastoid cell line (LCL) fibroblasts stably transfected with a plasmid encoding a single recombinant human HLA-DRB1 variant spanning common Caucasian alleles: DRB1*01:01, *03:01, *04:01, *07:01, *11:01, *13:01, and *15:01. LCL were split across 70 wells of a V-bottom 96-well plate and pulsed with 10 μg/mL peptide. We used 66 individual overlapping peptides (OLPs) spanning HIV Gag protein (clade B 2001 consensus-sequence) tested in singlet and had 4 negative control wells without peptide. Plates were incubated at 37°C and 5% CO_2 for 90 min and washed 6× to remove any unbound peptide. We then cultured 20,000 LCL with 100,000 CD8$^+$ T cells per well on a pre-coated interferon gamma (IFN-γ) plate. As a positive control, phytohemagglutinin (Sigma) was added at 1.8 μg/mL. The plates were incubated overnight at 37°C and 5% CO_2 and processed as previously described (Ranasinghe et al., 2013). We used the AID Elispot reader (Autoimmun Diagnostika GmbH) to determine the number of spot-forming cells per 100,000 CD8$^+$ T cells. A HLA-DR restriction was considered positive only if ≥3 times the mean background and also ≥3 times the standard deviation of negative control wells. A caveat is that each OLP was tested only once and some responses may be below the threshold for a "positive" result. To rectify this, any responses deemed above or close to the positive threshold were independently re-tested in quadruplicate.

HLA Class I and II Tetramers

Class II tetramers custom-manufactured by MBL International or made in collaboration with Dr. James Moon included DR11-Gag41 (DRB1*11:01-DRFYKTLRAEQASQEV) and DR01-Gag37 (DRB1*01:01-LNKIVRMYSPTSILD) conjugated to either PE or APC. Class I tetramers were made in collaboration with Dr. Soren Buus as described (Leisner et al., 2008). The class I tetramers included B57-KF11 (KAFSPEVIPMF), B08-EI8 (EIYKRWII), and B27-KK10 (KRWIILGLNK) in either PE or APC. Tetramers were incubated with whole PBMCs (25 min at 37°C, 5% CO_2) and then stained for viability and surface markers prior to fixation. Tetramer staining of hybridomas was conducted with the same protocol. Intracellular staining was used for Granzyme B and IFN-γ using the Cytofix/Cytoperm kit (BD PharMingen) according to the manufacturer's instructions. All fixed samples were analyzed on a LSRII flow cytometer (BD Biosciences) with FlowJo software (Treestar).

Functional Characterization of Class II-Restricted CD8$^+$ T Cells

To determine HIV-specific cytokine secretion in response to peptide-HLA-DR stimulus, whole PBMCs were incubated with 2 μg/mL of peptide pulsed onto LCL or left unstimulated in the presence of anti-HLA-DR (azide-free clone L243, Biolegend) to efficiently block HLA class II recognition. BFA and monensin were added to prevent cytokine secretion. IFN-γ secretion in response to peptide stimulus was measured from CD8$^+$ T cells. To confirm the restriction of the CD8$^+$ T cells, class I and class II tetramers were utilized. To determine proliferative capacity, PBMCs were stained with carboxyfluorescein diacetate succinimidyl ester (CFSE; Molecular Probes, Life Technologies) for 7 min at 37°C, then washed. Appropriate class I and class II peptides were added at 0.1 μg/mL to CFSE-labeled whole PBMCs for 7 days in RPMI 1640 medium in the absence of IL-2 at 37°C, 5% CO_2. PHA was used as a positive control and the absence of peptide stimulation was used as a negative control. After 7 days, cells were labeled with appropriate tetramer in APC together with antibodies to CD3, CD4, CD8, and CD25 and analyzed on a LSRII flow cytometer (BD Biosciences).

Chromium Release Assay

Chromium release assays were conducted with autologous targets (EBV-transformed B cell lines, CD4$^+$ T cells, and monocyte-derived macrophages), as described in Supplemental Experimental Procedures. Activated autologous CD4$^+$ T cells were infected with HIV NL4-3 by spinoculation at 800 × *g* for 1 hr at 37°C and cultured for 48 hr at 37°C and 5% CO_2. Autologous monocyte-derived macrophages were plated at 30,000 per well and VSV-G-pseudotyped SIV mac251 VLPs were added 3 hr prior to HIV challenge to abrogate host restriction factors and subsequently increase HIV infectivity. Macrophages were then infected with VSV-G-pseudotyped HIV NL4.3 expressing GFP by spinoculation at 800 × *g* for 1 hr at 37°C and cultured for 48 hr at 37°C and 5% CO_2. The CXCR4-utilizing HIV-1 laboratory strain NL4-3 and its VSV-G pseudotyped version were obtained from the AIDS Research and Reference Reagent Program, Division of AIDS, NIAID, NIH.

48 hr after HIV infection, CD4$^+$ T cells or macrophage target cells were labeled with chromium for 1 hr at 37°C and then washed 3 times. Tetramer-sorted CD8$^+$ T cells isolated from PBMCs or CD8$^+$ T cell clones were then added at the indicated effector-target ratios, and a standard 4–6 hr chromium release assay was performed as previously described (Chen et al., 2012).

Single-Cell RNA-Seq

Whole-transcriptome amplification of single cells in 96-well plates was performed with a modified SMART-Seq2 protocol, as described previously (Trombetta et al., 2014). Samples were sequenced on an Illumina NextSeq 500 instrument using either 30-bp paired-end reads or 150-bp single-end reads. RNA-seq reads were first trimmed using Trimmomatic (Bolger et al., 2014) and then aligned to the RefSeq hg38 transcriptome and genome using RSEM and TopHat. Considering only single-cell libraries in which we could reconstruct a productive TCR alignment, we excluded from further analysis genes and libraries with poor performance or coverage, leaving 205 cells and 3,274 genes. Out of the 205 cells with reconstructed TCR sequences, 30 were CD4$^+$ and 175 were CD8$^+$ by flow cytometry gating. The TPM expression of CD4, CD8A, and CD8B transcripts between the CD4$^+$ and CD8$^+$ cells was compared using Mann-Whitney-Wilcoxon test.

TCR α and β Chain Sequencing

In order to reconstruct CDR3 sequences from single-cell RNA-sequencing data, we developed TrapeS (TCR Reconstruction Algorithm for Paired-End Single cells), a software package for reconstruction of TCR sequences using short (~25 bp) single-cell paired-end RNA sequencing based on TopHat genomic alignments (Supplemental Experimental Procedures). TrapeS is available upon request.

TCR-Expressing T Cell Hybridomas

As previously described (White et al., 2000), an MSCV-derived retrovirus encoding GFP and the common TRBV2 V-domain fused to mouse Cβ and either the TRAV6 or TRAV26 domain fused mouse Cα was prepared. The virus was used to transduce a TCR$^-$ variant of the mouse T cell hybridoma that had been previously transduced to express human CD8α chain. T cells expressing high levels of TCR and CD8 were single cell cloned by FACS. 10^5 transduced T cell hybridomas were placed in 250 μL culture wells that had been either previously coated with an anti-mouse TCR Cβ Mab (H57-597) or contained 10^5 HLA-DR11-bearing LCL cells and various concentrations of the Gag41 peptide. After overnight culture at 37°C, the culture supernatants were assayed for IL-2 using the IL2-dependent cell line HT2.

Statistical Analysis

Paired t tests and Mann-Whitney-Wilcoxon test were used to compare different conditions, when each condition was tested in triplicate or greater. All p values are two-sided and $p < 0.05$ was considered significant. Statistical analysis and graphing were performed using GraphPad Prism 5.0 or R.

SUPPLEMENTAL INFORMATION

Supplemental Information includes six figures, two tables, and Supplemental Experimental Procedures and can be found with this article online at http://dx.doi.org/10.1016/j.immuni.2016.09.015.

AUTHOR CONTRIBUTIONS

S.R. was responsible for the concept and conduct of the study; S.R., P.A.L., and B.D.W. contributed to the experimental design and data analysis; S.R., P.A.L., D.Z.S., R.B.J., F.D., C.N., and P.J. performed the experiments; S.W.K. and A.K.S. conducted single-cell RNA-seq; M.B.C. and N.Y. computationally reconstructed TCR; G.M.C., F.C., J.W., A.M., and J.W.K. constructed TCR hybridomas and performed SPR; K.P. and T.M.A. conducted viral sequencing; H.S., D.E.K., and L.J.P. provided intellectual input and editorial comments; B.D.W. provided clinical samples and oversight of the project; S.R., P.A.L., and B.D.W. wrote the manuscript; and all authors contributed to revisions.

ACKNOWLEDGMENTS

We thank all study participants, as well as A. Sette and J. Sidney for the LCL, M.C. Carrington for HLA typing, and M. Nakayama for 5KC mouse hybridomas. This study was funded by the Bill & Melinda Gates Foundation Collaboration for AIDS Vaccine Discovery (CAVD) grant APP1108533 to L.J.P., B.D.W, and S.R. S.R. is also supported by a CFAR Developmental Award (NIH/NIAID 5P30AI060354). P.A.L. is supported by a HHMI International Student Research Fellowship. D.E.K. is supported by a Research Scholar Career Award of the Quebec Health Research Fund. T.M.A. and K.P. are supported by NIAID grant AI104715. S.W.K. is supported by a NSF Graduate Research Fellowship. A.K.S. is supported by the Ragon Institute, the Searle Scholars Program, the Beckman Young Investigator Program, the NIH New Innovator Award (DP2 OD020839), and NIH U24 AI11862-01 from NIAID. L.J.P. and OHSU have a significant financial interest in Vir Bio, Inc., a company that may have a commercial interest in the results of this research and technology. The potential individual and institutional conflicts of interest have been reviewed and are being managed by OHSU. The content of this publication does not necessarily reflect the views or policies of the Department of Health and Human Services, U.S. Army, or Department of Defense, nor does mention of trade names, commercial products, or organizations imply endorsement by the U.S. Government. The funders had no role in study design, data collection and interpretation, or the decision to submit the work for publication.

Received: June 3, 2016
Revised: August 9, 2016
Accepted: August 15, 2016
Published: October 18, 2016

REFERENCES

Almeida, J.R., Price, D.A., Papagno, L., Arkoub, Z.A., Sauce, D., Bornstein, E., Asher, T.E., Samri, A., Schnuriger, A., Theodorou, I., et al. (2007). Superior control of HIV-1 replication by CD8+ T cells is reflected by their avidity, polyfunctionality, and clonal turnover. J. Exp. Med. *204*, 2473–2485.

Benati, D., Galperin, M., Lambotte, O., Gras, S., Lim, A., Mukhopadhyay, M., Nouël, A., Campbell, K.A., Lemercier, B., Claireaux, M., et al. (2016). Public T cell receptors confer high-avidity CD4 responses to HIV controllers. J. Clin. Invest. *126*, 2093–2108.

Bolger, A.M., Lohse, M., and Usadel, B. (2014). Trimmomatic: a flexible trimmer for Illumina sequence data. Bioinformatics *30*, 2114–2120.

Carrington, M., and Walker, B.D. (2012). Immunogenetics of spontaneous control of HIV. Annu. Rev. Med. *63*, 131–145.

Chen, H., Ndhlovu, Z.M., Liu, D., Porter, L.C., Fang, J.W., Darko, S., Brockman, M.A., Miura, T., Brumme, Z.L., Schneidewind, A., et al. (2012). TCR clonotypes modulate the protective effect of HLA class I molecules in HIV-1 infection. Nat. Immunol. *13*, 691–700.

Emu, B., Sinclair, E., Hatano, H., Ferre, A., Shacklett, B., Martin, J.N., McCune, J.M., and Deeks, S.G. (2008). HLA class I-restricted T-cell responses may contribute to the control of human immunodeficiency virus infection, but such responses are not always necessary for long-term virus control. J. Virol. *82*, 5398–5407.

Fellay, J., Shianna, K.V., Ge, D., Colombo, S., Ledergerber, B., Weale, M., Zhang, K., Gumbs, C., Castagna, A., Cossarizza, A., et al. (2007). A whole-genome association study of major determinants for host control of HIV-1. Science *317*, 944–947.

Hansen, S.G., Sacha, J.B., Hughes, C.M., Ford, J.C., Burwitz, B.J., Scholz, I., Gilbride, R.M., Lewis, M.S., Gilliam, A.N., Ventura, A.B., et al. (2013). Cytomegalovirus vectors violate CD8+ T cell epitope recognition paradigms. Science *340*, 1237874.

Hansen, S.G., Wu, H.L., Burwitz, B.J., Hughes, C.M., Hammond, K.B., Ventura, A.B., Reed, J.S., Gilbride, R.M., Ainslie, E., Morrow, D.W., et al. (2016). Broadly targeted $CD8^+$ T cell responses restricted by major histocompatibility complex E. Science *351*, 714–720.

Heemskerk, M.H., de Paus, R.A., Lurvink, E.G., Koning, F., Mulder, A., Willemze, R., van Rood, J.J., and Falkenburg, J.H. (2001). Dual HLA class I and class II restricted recognition of alloreactive T lymphocytes mediated by a single T cell receptor complex. Proc. Natl. Acad. Sci. USA *98*, 6806–6811.

Hirosawa, T., Torikai, H., Yanagisawa, M., Kamei, M., Imahashi, N., Demachi-Okamura, A., Tanimoto, M., Shiraishi, K., Ito, M., Miyamura, K., et al. (2011). Mismatched human leukocyte antigen class II-restricted $CD8^+$ cytotoxic T cells may mediate selective graft-versus-leukemia effects following allogeneic hematopoietic cell transplantation. Cancer Sci. *102*, 1281–1286.

Horton, H., Frank, I., Baydo, R., Jalbert, E., Penn, J., Wilson, S., McNevin, J.P., McSweyn, M.D., Lee, D., Huang, Y., et al. (2006). Preservation of T cell proliferation restricted by protective HLA alleles is critical for immune control of HIV-1 infection. J. Immunol. *177*, 7406–7415.

Iglesias, M.C., Almeida, J.R., Fastenackels, S., van Bockel, D.J., Hashimoto, M., Venturi, V., Gostick, E., Urrutia, A., Wooldridge, L., Clement, M., et al. (2011). Escape from highly effective public CD8+ T-cell clonotypes by HIV. Blood *118*, 2138–2149.

Kaslow, R.A., Carrington, M., Apple, R., Park, L., Muñoz, A., Saah, A.J., Goedert, J.J., Winkler, C., O'Brien, S.J., Rinaldo, C., et al. (1996). Influence of combinations of human major histocompatibility complex genes on the course of HIV-1 infection. Nat. Med. *2*, 405–411.

Kiepiela, P., Leslie, A.J., Honeyborne, I., Ramduth, D., Thobakgale, C., Chetty, S., Rathnavalu, P., Moore, C., Pfafferott, K.J., Hilton, L., et al. (2004). Dominant influence of HLA-B in mediating the potential co-evolution of HIV and HLA. Nature *432*, 769–775.

Kiepiela, P., Ngumbela, K., Thobakgale, C., Ramduth, D., Honeyborne, I., Moodley, E., Reddy, S., de Pierres, C., Mncube, Z., Mkhwanazi, N., et al. (2007). CD8+ T-cell responses to different HIV proteins have discordant associations with viral load. Nat. Med. *13*, 46–53.

Leisner, C., Loeth, N., Lamberth, K., Justesen, S., Sylvester-Hvid, C., Schmidt, E.G., Claesson, M., Buus, S., and Stryhn, A. (2008). One-pot, mix-and-read peptide-MHC tetramers. PLoS ONE *3*, e1678.

Mason, D. (1994). Allelic exclusion of alpha chains in TCRs. Int. Immunol. *6*, 881–885.

Matechak, E.O., Killeen, N., Hedrick, S.M., and Fowlkes, B.J. (1996). MHC class II-specific T cells can develop in the CD8 lineage when CD4 is absent. Immunity *4*, 337–347.

Mendoza, D., Royce, C., Ruff, L.E., Ambrozak, D.R., Quigley, M.F., Dang, T., Venturi, V., Price, D.A., Douek, D.C., Migueles, S.A., and Connors, M. (2012). HLA B*5701-positive long-term nonprogressors/elite controllers are not distinguished from progressors by the clonal composition of HIV-specific CD8+ T cells. J. Virol. *86*, 4014–4018.

Migueles, S.A., Sabbaghian, M.S., Shupert, W.L., Bettinotti, M.P., Marincola, F.M., Martino, L., Hallahan, C.W., Selig, S.M., Schwartz, D., Sullivan, J., and Connors, M. (2000). HLA B*5701 is highly associated with restriction of virus replication in a subgroup of HIV-infected long term nonprogressors. Proc. Natl. Acad. Sci. USA *97*, 2709–2714.

Mizuochi, T., Tentori, L., Sharrow, S.O., Kruisbeek, A.M., and Singer, A. (1988). Differentiation of Ia-reactive CD8+ murine T cells does not require Ia engagement. Implications for the role of CD4 and CD8 accessory molecules in T cell differentiation. J. Exp. Med. *168*, 437–442.

Neefjes, J., Jongsma, M.L., Paul, P., and Bakke, O. (2011). Towards a systems understanding of MHC class I and MHC class II antigen presentation. Nat. Rev. Immunol. *11*, 823–836.

Pearce, E.L., Shedlock, D.J., and Shen, H. (2004). Functional characterization of MHC class II-restricted CD8+CD4- and CD8-CD4- T cell responses to infection in CD4-/- mice. J. Immunol. *173*, 2494–2499.

Pereyra, F., Jia, X., McLaren, P.J., Telenti, A., de Bakker, P.I., Walker, B.D., Ripke, S., Brumme, C.J., Pulit, S.L., Carrington, M., et al.; International HIV Controllers Study (2010). The major genetic determinants of HIV-1 control affect HLA class I peptide presentation. Science *330*, 1551–1557.

Ranasinghe, S., and Walker, B.D. (2013). Programming CMV for vaccine vector design. Nat. Biotechnol. *31*, 811–812.

Ranasinghe, S., Flanders, M., Cutler, S., Soghoian, D.Z., Ghebremichael, M., Davis, I., Lindqvist, M., Pereyra, F., Walker, B.D., Heckerman, D., and Streeck, H. (2012). HIV-specific CD4 T cell responses to different viral proteins have discordant associations with viral load and clinical outcome. J. Virol. *86*, 277–283.

Ranasinghe, S., Cutler, S., Davis, I., Lu, R., Soghoian, D.Z., Qi, Y., Sidney, J., Kranias, G., Flanders, M.D., Lindqvist, M., et al. (2013). Association of HLA-DRB1-restricted $CD4^{+}$ T cell responses with HIV immune control. Nat. Med. *19*, 930–933.

Riou, C., Burgers, W.A., Mlisana, K., Koup, R.A., Roederer, M., Abdool Karim, S.S., Williamson, C., and Gray, C.M. (2014). Differential impact of magnitude, polyfunctional capacity, and specificity of HIV-specific CD8+ T cell responses on HIV set point. J. Virol. *88*, 1819–1824.

Rist, M., Smith, C., Bell, M.J., Burrows, S.R., and Khanna, R. (2009). Cross-recognition of HLA DR4 alloantigen by virus-specific CD8+ T cells: a new paradigm for self-/nonself-recognition. Blood *114*, 2244–2253.

Schwartz, O., Maréchal, V., Le Gall, S., Lemonnier, F., and Heard, J.M. (1996). Endocytosis of major histocompatibility complex class I molecules is induced by the HIV-1 Nef protein. Nat. Med. *2*, 338–342.

Scriba, T.J., Purbhoo, M., Day, C.L., Robinson, N., Fidler, S., Fox, J., Weber, J.N., Klenerman, P., Sewell, A.K., and Phillips, R.E. (2005). Ultrasensitive detection and phenotyping of CD4+ T cells with optimized HLA class II tetramer staining. J. Immunol. *175*, 6334–6343.

Shimizu, T., and Takeda, S. (1997). CD8 T cells from major histocompatibility complex class II-deficient mice respond vigorously to class II molecules in a primary mixed lymphocyte reaction. Eur. J. Immunol. *27*, 500–508.

Southwood, S., Sidney, J., Kondo, A., del Guercio, M.F., Appella, E., Hoffman, S., Kubo, R.T., Chesnut, R.W., Grey, H.M., and Sette, A. (1998). Several common HLA-DR types share largely overlapping peptide binding repertoires. J. Immunol. *160*, 3363–3373.

Suzuki, H., Eshima, K., Takagaki, Y., Hanaoka, S., Katsuki, M., Yokoyama, M., Hasegawa, T., Yamazaki, S., and Shinohara, N. (1994). Origin of a T cell clone with a mismatched combination of MHC restriction and coreceptor expression. J. Immunol. *153*, 4496–4507.

Trombetta, J.J., Gennert, D., Lu, D., Satija, R., Shalek, A.K., and Regev, A. (2014). Preparation of single-cell RNA-seq libraries for next generation sequencing. Curr. Protoc. Mol. Biol. *107*, 1–17.

Tyznik, A.J., Sun, J.C., and Bevan, M.J. (2004). The CD8 population in CD4-deficient mice is heavily contaminated with MHC class II-restricted T cells. J. Exp. Med. *199*, 559–565.

van der Merwe, P.A., and Davis, S.J. (2003). Molecular interactions mediating T cell antigen recognition. Annu. Rev. Immunol. *21*, 659–684.

White, J., Kappler, J., and Marrack, P. (2000). Production and characterization of T cell hybridomas. Methods Mol. Biol. *134*, 185–193.

Immunity, Volume *45*

Supplemental Information

Antiviral CD8$^+$ T Cells Restricted by Human Leukocyte Antigen Class II Exist during Natural HIV Infection and Exhibit Clonal Expansion

Srinika Ranasinghe, Pedro A. Lamothe, Damien Z. Soghoian, Samuel W. Kazer, Michael B. Cole, Alex K. Shalek, Nir Yosef, R. Brad Jones, Faith Donaghey, Chioma Nwonu, Priya Jani, Gina M. Clayton, Frances Crawford, Janice White, Alana Montoya, Karen Power, Todd M. Allen, Hendrik Streeck, Daniel E. Kaufmann, Louis J. Picker, John W. Kappler, and Bruce D. Walker

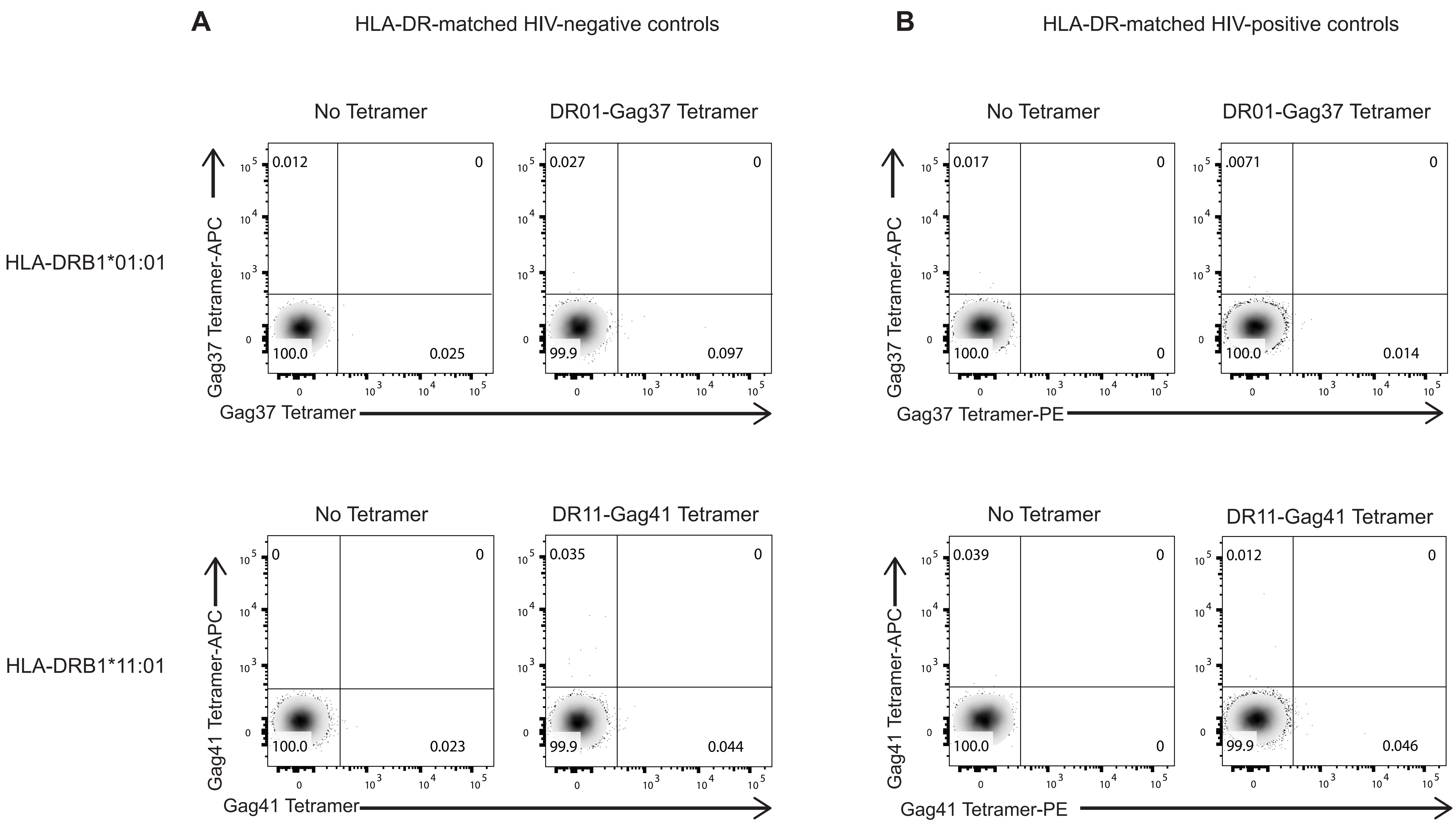

Figure S1. Related to Figure 2. HLA-DR tetramer validation in HIV-negative and HIV-positive subjects.
Representative FACS plot of HLA class II tetramer staining of fresh PBMCs in HLA-matched HIV-negative (A) and HIV-positive (B) subjects. Populations shown are gated on CD3+CD8+CD4-CD19-CD14-CD56- live lymphocyte singlets. Bulk CD8+ T cells are shown in the absence and presence of tetramer.

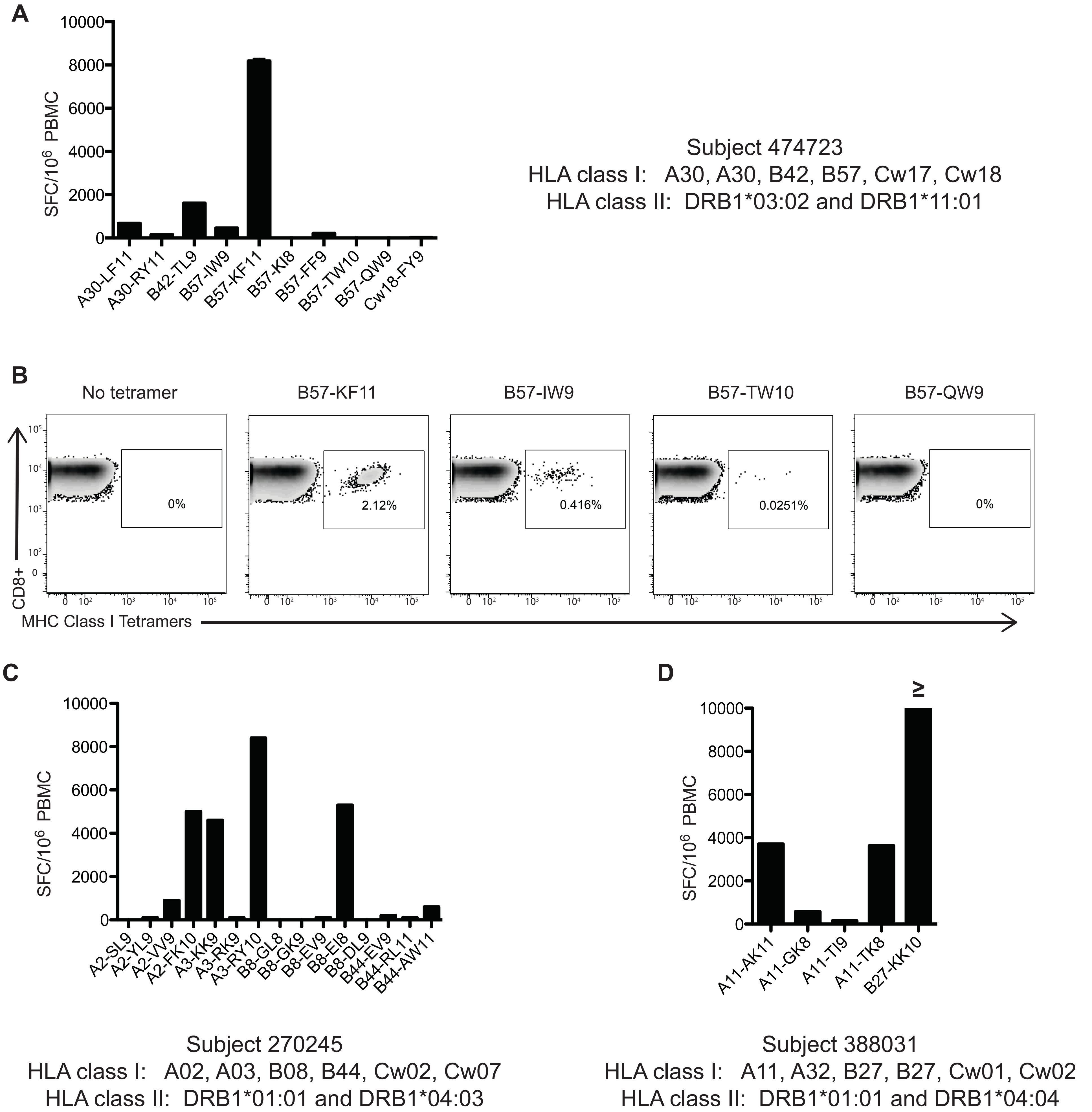

Figure S2. Related to Figure 1 and 2. Epitope-targeting of HLA class I- versus class II-restricted CD8+ T cells.
(A) Summary graph of HLA class I-restricted Gag-specific CD8+ T cells in subject 474723 as measured in a standard IFN-γ ELISPOT assay. Known optimal peptides matching the subject's HLA class I typing were added to whole PBMC. Error bars with standard deviation (SD) is shown (B) Representative FACS plots of ex-vivo tetramer positive CD8+ T cells in subject 474723 to immunodominant HLA-B*57 epitopes KF11, IW9, TW10 and QW9. (C) Summary graph of HLA class I-restricted Gag-specific CD8+ T cells in subject 270245 as measured in a standard IFN-γ ELISPOT assay. Known optimal peptides matching the subject's HLA class I were added to whole PBMC. (D) Summary graph of HLA class I-restricted Gag-specific CD8+ T cells in subject 388031 as measured in a standard IFN-γ ELISPOT assay. Known optimal peptides matching the subject's HLA class I typing were added to whole PBMC. The B*27-KK10 response was greater than the threshold of accurate detection (9999 SFU/106) as represented by "≥".

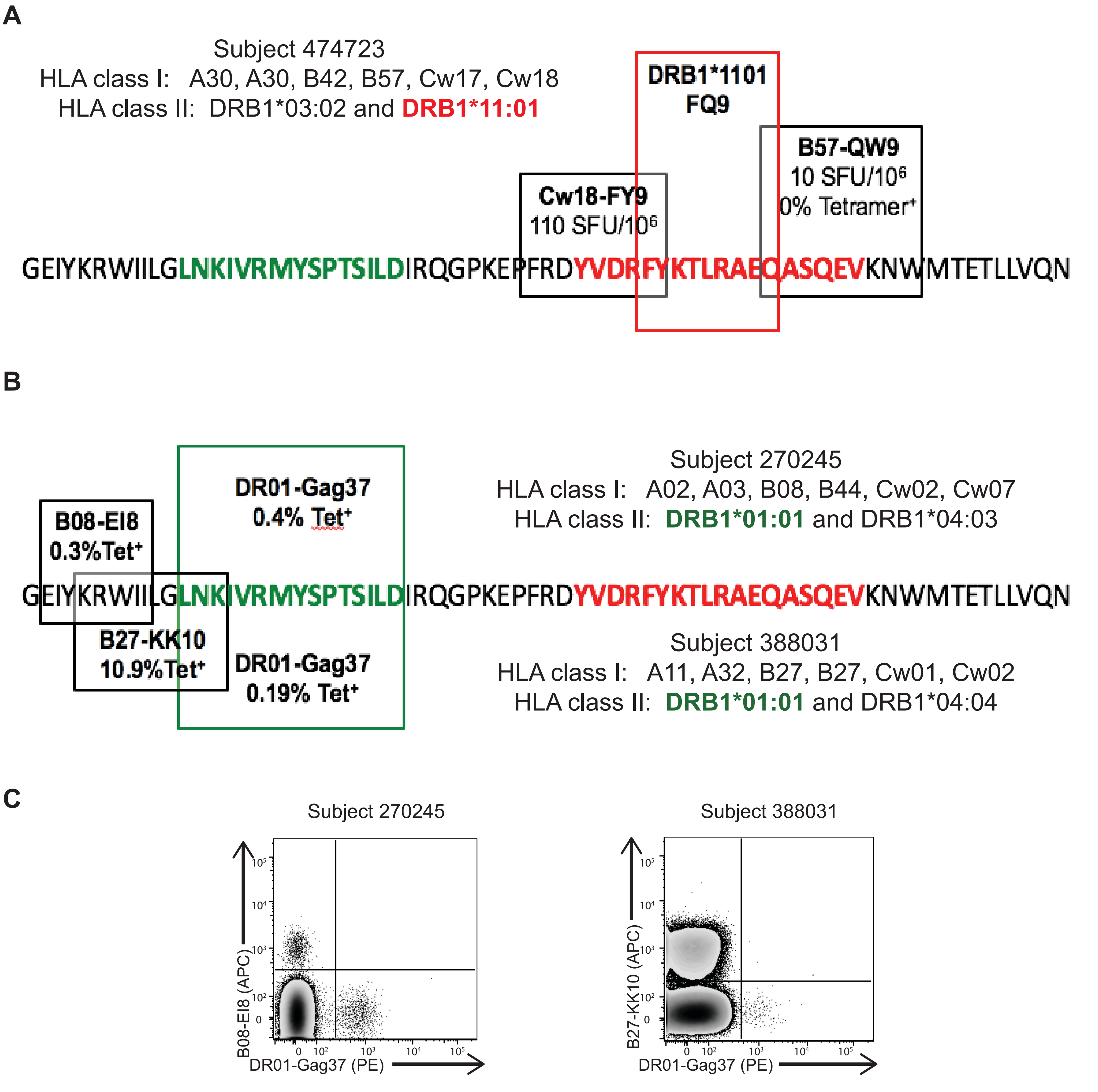

Figure S3. Related to Figure 1 and 2. Epitope-mapping of class I- versus class II-restricted CD8+ T cells.
(A) Schematic of a partial Gag p24 sequence highlighting the Gag41 peptide residues in red text are shown and the predicted core peptide FA10 in a red box for subject 473723. HLA class I epitopes B*57-QW9 and Cw*18-FY9 is shown in black boxes, with values corresponding to standard IFN-γ ELISPOT and tetramer staining. (B) Schematic of a partial Gag p24 sequence highlighting the Gag37 peptide residues in green text and values corresponding to DR01-Gag37 tetramer staining for subjects 270245 and 388031 in a green box. For subject 270245, value corresponding to B*08-EI8 tetramer staining is shown in a black box. For subject 388031, value corresponding to B*27-KK10 tetramer staining is shown in a black box. For reference, the Gag41 sequence is highlighted in red text. (C) Representative FACS plot denoting percentage frequency of ex-vivo tetramer positive CD8+ T cells to DR01-Gag37 and B*08-EI8 in subject 270245, and DR01-Gag37 and B*27-KK10 in subject 388031, as determined by dual staining with HLA class I (APC) and II (PE) tetramers.

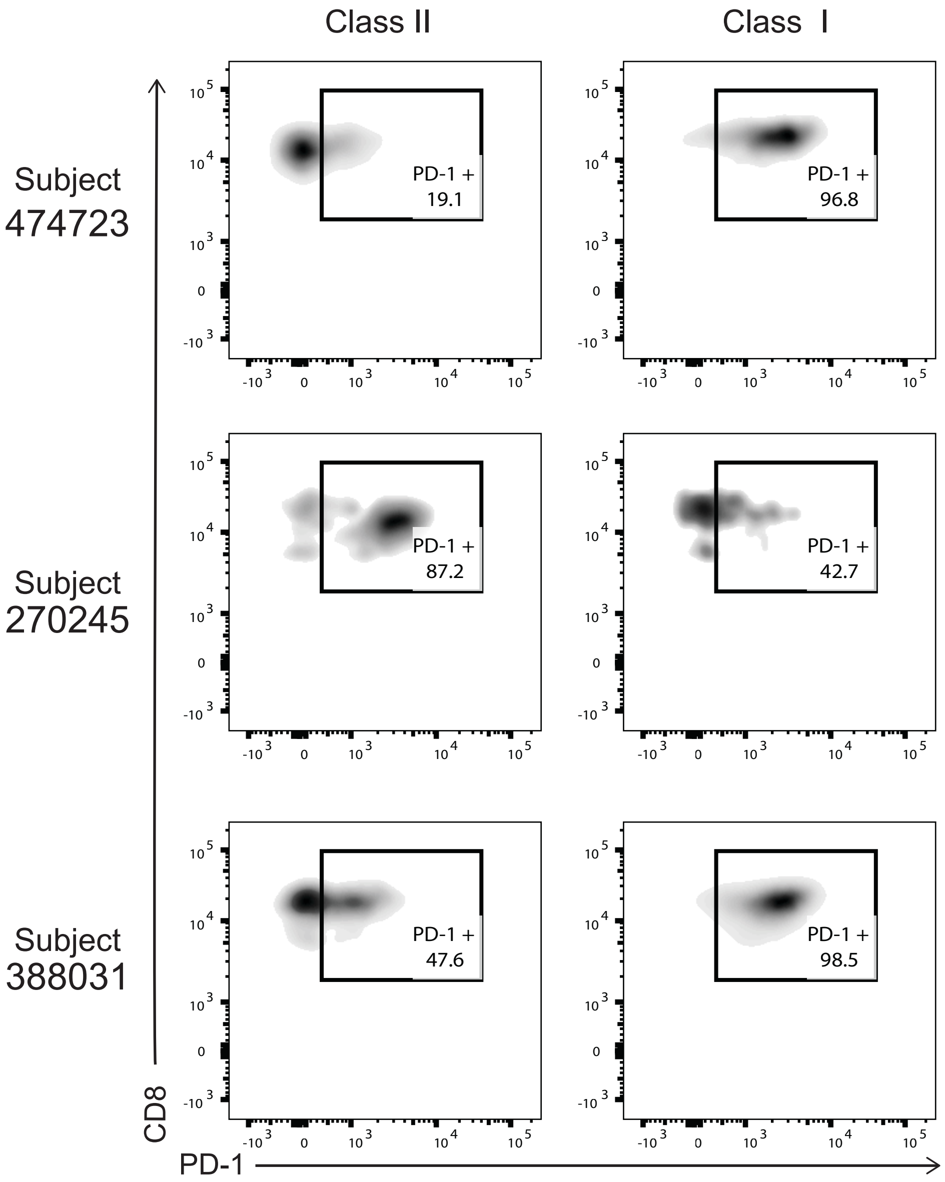

Figure S4. Related to Figure 3. PD-1 expression on class I- and class II-restricted CD8+ T cells.
Representative FACS plots of surface PD-1 expression on tetramer positive HLA class I and class II-restricted CD8+ T cells. Populations shown are gated on tetramer+ CD3+ CD8+ CD4- CD19- CD14- CD56- live lymphocyte singlets.

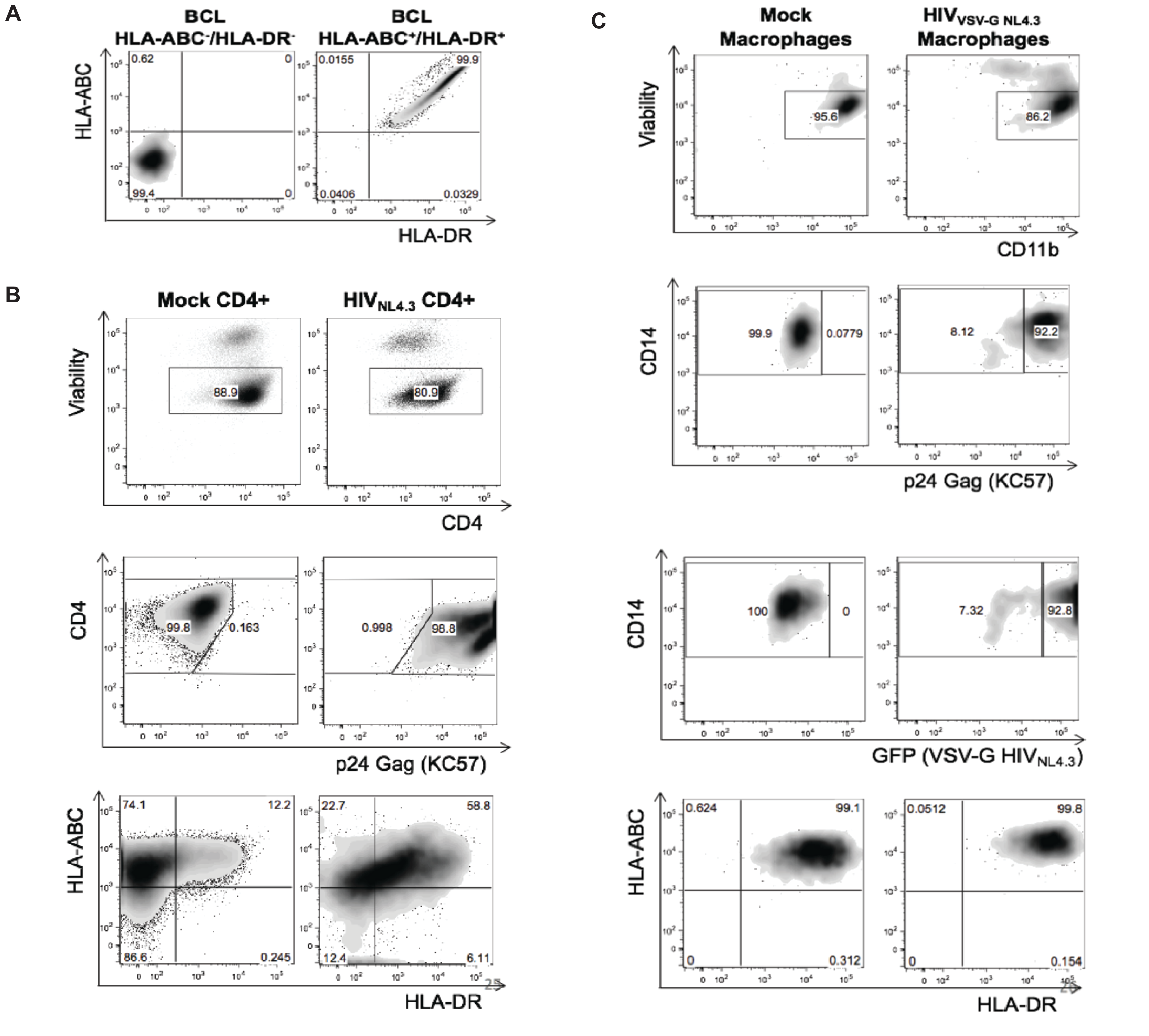

Figure S5. Related to Figure 5. HIV infection and HLA expression of Target cells.
(A) EBV-transformed B cell line, (B) CD4+ T cells, and (C) monocyte-derived macrophages from subject 474723. Representative FACS plots denoting surface HLA class I ABC and HLA-DR expression on autologous target cells using W6/32 and L432 antibodies, respectively. The proportion of intracellular Gag p24 positive CD4+ T cells was analyzed by flow cytometry 48 hrs post-infection using KC57 antibody. The CD4+ T cells were blasted with CD3+CD28+ dynabeads 3 days prior to infection by spinoculation with HIV NL4.3. The proportion of GFP positive and intracellular Gag p24 KC57 antibody positive monocyte derived macrophages (CD14+ CD11b+) was analyzed 48 hrs post-infection with VSV-G psuedotyped HIV NL4.3.

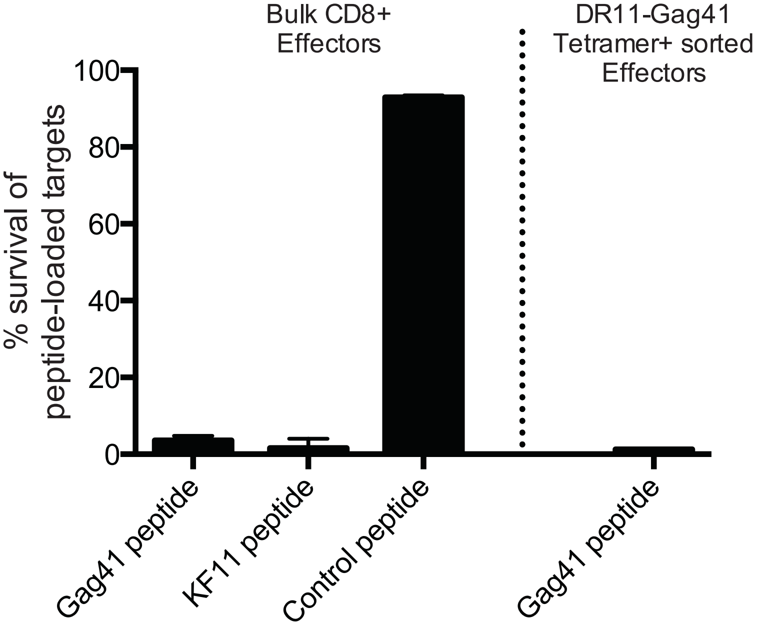

Figure S6. Related to Figure 5. Specific elimination of peptide-loaded target cells in a modified VITAL assay.

Summary graph of ex-vivo bulk CD8+ T cells and tetramer-sorted DR11-Gag41 positive cells from subject 474723 tested in a modified 36 hr VITAL assay. Ex-vivo effector cells were derived from fresh blood; processed and isolated within 12hrs. Bulk CD8+ T cells were isolated using Miltenyi CD8 MACS beads. DR11-Gag41 positive CD8+ T cells were tetramer stained and isolated by FACS. Autologous EBV-transformed BCL were used as target cells, in which half of the autologous BCL were pulsed with peptide and labeled with CFSE (Carboxyfluorescein succinimidyl ester), and the other half remained without peptide and were labeled with CTV (Cell Trace Violet), prior to co-culture with the tetramer sorted effectors in duplicate. After 36 hours, the co-culture was stained and analyzed by flow cytometry. Error bars with standard deviation shows the percentage survival of peptide-loaded cells (CFSE+).

HLA-DRB1 expression	No. of **HIV Controllers** screened by HLA-DRB1 ELISpot	No. of **HIV Controllers** screened by DR11-Gag41 tetramer	No. of **HIV Controllers** screened by DR01-Gag37 tetramer	No. of HIV Controllers with detectable DRB1-restricted CD8+ T cells
DRB1*01:01	25	-	25*	2
DRB1*04:01	18	-	-	0
DRB1*07:01	27	-	-	0
DRB1*11:01	31	68[#]	-	1
TOTAL	**101**	**68**	**25**	**3**

HLA-DRB1 expression	No. of **HIV Progressors** screened by HLA-DRB1 ELISpot	No. of **HIV Progressors** screened by DR11-Gag41 tetramer	No. of **HIV Progressors** screened by DR01-Gag37 tetramer	No. of HIV Progressors with detectable DRB1-restricted CD8+ T cells
DRB1*01:01	4	-	4*	0
DRB1*03:01	3	-	-	0
DRB1*07:01	7	-	-	0
DRB1*11:01	8	8[#]	-	0
DRB1*13:01	3	-	-	0
DRB1*15:01	3	-	-	0
TOTAL	**28**	**8**	**4**	**0**

Table S1. Related to Figure 1 and 2. Cohort of HIV-infected individuals screened for $CD8^+$ T-cell responses restricted by HLA-DR. A total of 129 HIV-infected individuals were screened for HLA-DR-restricted $CD8^+$ T-cell responses by IFN-γ Elispot and by flow cytometry using HLA class II tetramers, as depicted in the table. [#]DRB1*11 subjects screened by tetramer included all DR11 subjects previously screened by HLA-DRB1 Elispot. *DRB1*01 subjects screened by tetramer included all DR01 subjects previously screened by HLA-DRB1 Elispot

Ragon Identifier	474723	270245	388031
HIV status#	Viremic Controller	Viremic Controller	Elite Controller
Viral Load (RNA copies/ml)#	136	20	<75
CD4 count (cells/uL)#	1137	684	1100
HIV Diagnosis Year	1997	1982	2004
Enrolled in the Cohort	2007 until present	2005 until present	2005 until 2013
ART	1997-2003,<br>off therapy 2003-2015,<br>Restarted therapy Jan 2016	1987-2000,<br>off-therapy from 2000 to present	Treatment-naive
Year of Birth	1964	1947	1966
Gender	Male	Male	Male
Race	Black or African American	White	White
HIV Risk	Blood products	MSM	MSM
Known co-infections#	HBV	TBD	TBD
HLA class I alleles	A*30:01, A*30:02,<br>B*42:01, B*57:03,<br>Cw17, Cw18	A*02:01, A*03:01,<br>B*08:1, B44*05,<br>Cw*02:02, Cw*07:01	A*11:01, A*32:01,<br>B*27:05, B*27:05,<br>Cw*01:02, Cw*02:02
HLA class II alleles	DRB1*03:02,<br>DRB1*11:01,<br>DPB*01:01,<br>DPB*39:01,<br>DQB*04:02,<br>DQB*05:01,	DRB1*01:01,<br>DRB1*04:03	DRB1*01:01,<br>DRB1*04:01,<br>DPB*04:01,<br>DPB*:04:01,<br>DQB*03:02,<br>DQB*05:01,

Table S2. Related to Figure 2. Clinical characteristics of study subjects with detectable HLA class II-restricted $CD8^+$ T cells. The clinical characteristics for study subjects 474723, 270245, and 388031 are depicted in the table. The former two subjects are currently enrolled, however, 388031 left the cohort in 2013 with almost no sample availability remaining. Subject 474723 started antiretroviral therapy in 2016. # Information provided for principal date of assays utilizing fresh or frozen PBMC. MSM: Men who have sex with men. HBV: Hepatitis B virus

SUPPLEMENTAL EXPERIMENTAL PROCEDURES

Subjects

All study subjects gave informed consent and IRB approval was obtained from the Massachusetts General Hospital institutional regulatory board (IRB). 101 individuals recruited in this study were 'HIV Controllers', defined as HIV infected individuals who spontaneously control HIV infection in the absence of antiretroviral therapy for greater than 1 year. These HIV controllers included 40 'Elite Controllers' with viral loads of below 50 HIV RNA copies/ml for greater than 1 year, and 61 'Viremic Controllers' with viral loads of between >50 <2,000 HIV RNA copies/ml for greater than 1 year. Additionally, 28 treatment-naive HIV progressors with viral loads of greater than 2,000 HIV RNA copies/ml were screened. All subjects were chosen based on delineation of their HLA class II DRB1 alleles (with all individuals selected upon expression of one or more common DRB1 alleles spanning *01:01, *03:01, *04:01, *07:01, *11:01, *13:01 and *15:01 and availability of frozen peripheral blood mononuclear cell (PBMC) samples for HLA-DR CD8 Elispots and further functional characterization (Tables S1 and S2).

Human leukocyte antigen typing.

High resolution four-digit HLA genotyping was performed by sequence-specific PCR in accordance with standard procedures. Briefly, HLA class I–encoding genes were amplified by PCR with primers spanning exons 2 and 3, and HLA class II DRB1–encoding genes were identified by PCR amplification and sequencing of exon 2. ASSIGN 3.5 software developed by Conexio Genomics was used to interpret the sequencing results.

Peptide synthesis

Overlapping Peptides (OLPs) corresponding to HIV-1 clade B consensus 2001 for Gag protein were synthesized at the MGH Peptide Core Facility on an automated peptide synthesizer using F-moc technology. In addition, N- and C-terminal truncated peptides were synthesized for Gag41.

Elispot with whole PBMC

Screening for class I-restricted HIV-specific $CD8^+$ T cell responses was conducted using a standardized IFN-γ Elispot assay with whole PBMC, as previously described (Ranasinghe et al., 2012). In brief, whole PBMC was co-cultured individually with 10 μg/mL optimally defined class I epitopes (concordant with HLA class I typing for that individual). Input cell numbers were 100,000 whole PBMC per well and the plates were incubated overnight at 37 °C and 5% CO_2. Responses were regarded as positive if they had at least 3 times the mean background and ≥3 times the standard deviation of the negative control wells; positive responses also had to be at least 50 SFC/10^6 PBMCs.

Epitope fine-mapping

For fine-mapping analysis, $CD8^+$ T cells enriched from whole PBMC by Miltenyi $CD8^+$ MACS MicroBeads were isolated. The IFN-γ responses of $CD8^+$ T cell populations against serial truncations of Gag41 (YVDRFYKTLRAEQASQEV) restricted by DRB1*11:01 were tested. Each peptide was tested at 20 μM including, with serial truncations from the N and C termini, presented by the restricting HLA-DRB1–expressing L cell lines. Enriched $CD8^+$ T cells were co-cultured at 100,000 cells with 20,000 LCL on a modified 'HLA-DR CD8 Elispot'.

Generation of $CD8^+$ T cell clones

Whole PBMC were thawed and rested for 2 hours, with half of the PBMC then pulsed with the respective peptide of interest for 1 hour at 37 ºC, 5% CO2. After pulsing, the PBMC were washed to remove free peptide and then cultured in 60 wells of a 96-well round-bottom plate in RPMI 1640 medium containing 50 U/ml of recombinant IL-2. After approximately 2 weeks of culture, the whole PBMC TCL were then tested using the respective HLA class I or II tetramer to ascertain the percentage specificity of the population. Peptide-specific T cells were isolated using an IFN-γ secretion assay (Miltenyi), as per the manufacture's protocol. Isolated IFN-γ-positive T cells were cultured with irradiated allogeneic PBMC and CD3-specific antibody as a T cell proliferation stimulus for approximately 2 weeks and then limited dilution cloning was conducted, as previously described (Chen et al., 2012). Developing epitope-specific $CD8^+$ T cell clones were further tested separately by chromium release assays to their respective peptide, and by tetramer staining to confirm their $CD8^+$ T cell specificity. Cloned $CD8^+$ T cells were maintained by restimulation every 14 to 21 days with an anti-CD3 mAb and irradiated allogeneic PBMC in RPMI 1640 medium containing 50 U/ml of recombinant IL-2, as previously described (Ranasinghe et al., 2011) (Jones et al., 2012)

Generation of autologous targets cells for killing assays

EBV-transformed B cell lines. 10 million frozen PBMC were thawed and resuspended in 1mL of RMPI, 1.5mL of fetal bovine serum (FBS), and 1.5mL of unconcentrated supernatant of Epstein-Barr virus. Cyclosporine A (sigma) was added in a 1 µg/ml concentration. Cell were cultured for 6 to 8 weeks at 37 °C and 5% CO2

Activated CD4+ T cells. CD4+ T cell were isolated from frozen PBMC using CD4 Macs Beads (Miltenyi) and blasted with human T-activator CD3/CD28 dynabeads (ThermoFisher) for three days at 37 °C and 5% CO2

Monocyte-derived macrophages. CD14+ cells were isolated from frozen PBMC using CD14 enrichment EasySep (STEM Cell) and cultured for 6 days at 37 °C and 5% CO2 to differentiate into macrophages (CD14+ CD11b+)

Infection assessment of target cells in killing assays

To assess infectivity, target cells were surface stained with either anti-CD4 or anti-CD11b antibody, and intracellular stained with anti-p24 antibody, KC57-RD1 (Beckman Coulter). The VSV-G pseudotyped HIV NL4-3 was obtained from the NIH AIDS Reagent Program, Division of AIDS, NIAID, NIH: pNL4-3-deltaE-EGFP (Cat# 11100) from Drs. Haili Zhang, Yan Zhou, and Robert Siliciano.

Vital Assay

A modified VITAL assay (Hermans et al., 2004) was conducted with autologous EBV-transformed B cell lines. BCL were stained either with carboxyfluorescein diacetate succinimidyl ester (CFSE; Molecular Probes, Life Technologies, USA) or cell trace violet (CTV; Molecular Probes, Life Technologies) for 7 min at 37°C and then washed. 10 µg/mL of appropriate peptide was added separately to CFSE labeled cells, while CTV labeled cells remained without peptide, for 1 hour at 37 ºC, 5% CO_2. After several washes, 25,000 peptide$^+$ CFSE$^+$ cells were mixed with 25,000 peptide$^-$ CTV$^+$ cells to give a total of 50,000 targets per well. The target cells were then co-cultured with fresh Gag41 Tetramer-sorted CD8$^+$ T cells or bulk CD8$^+$ T cells, at a 1:1 E:T ratio. The co-cultures were incubated for 36 hours at 37 ºC, 5% CO_2. After 36 hours, the co-cultures were surface stained with anti-CD19, -CD3, -CD4, -CD8 antibodies, and a viability marker. Compensation was performed (including CFSE and CTV) and the fixed samples were analyzed on a LSRII flow cytometer (BD Biosciences). Analysis focused on the percentage of viable peptide-loaded CFSE$^+$ cells vs. CTV$^+$ cells, which lacked the cognate peptide.

Viral Sequencing

Genomic DNA was isolated from frozen PBMC as previously described (Miura et al., 2008). In brief, genomic DNA was isolated using the Qiagen DNA blood mini kit (Qiagen Inc., Valencia, CA) and HIV-1 Gag was amplified using nested reverse transcriptase PCR (RT-PCR). The primer sequences are available upon request. PCR products were prepared for sequencing on the 454 Genome Sequencer FLX Titanium (Roche) using standard protocols with modifications as previously described (Henn et al., 2012).

Single-Cell RNA-seq

Whole Transcriptome amplification (WTA). WTA of single cells in 96 well plates was performed with a modified SMART-Seq2 protocol, as described previously (Trombetta et al., 2014), with Maxima Reverse Transcriptase (Life Technologies) used in place of superscript II. WTA products were then cleaned with Agencourt XP DNA beads (DNA SPRI) and 80% ethanol (Beckman Coulter) and Illumina sequencing libraries were prepared using Nextera XT (Illumina). The 96 samples in each plate were pooled together, and cleaned with two 0.9x DNA SPRIs (Beckman Coulter). Library quality was assessed with a high sensitivity DNA chip (Agilent) and quantified with a high sensitivity dsDNA Quant Kit (Life Technologies). Samples were sequenced on an Illumina NextSeq 500 instrument using either 30bp paired-end reads or 150bp single-end reads.

Single-Cell RNA-Seq Preprocessing. RNA-seq reads were first trimmed using Trimmomatic (Bolger et al.,2014). Trimmed reads were aligned to the RefSeq hg38 genome and transcriptome (GRCh38.2) using TopHat (Trapnell et al., 2009) and Bowtie2 (Langmead and Salzberg, 2012) respectively. The resulting transcriptome alignments were processed by RSEM to estimate the abundance (TPM) of RefSeq transcripts (Li and Dewey, 2011).

Sample Filtering and Normalization. Considering only single-cell libraries in which we could reconstruct a productive TCR alignment (see below), we excluded from further analysis libraries with poor values for total number of reads (< 25000 reads), the percentage of aligned reads (< 10% aligned), or the percentage of detected transcripts (< 20% detected). All transcripts with lower than 10 TPM expression in more than 85% of samples were removed from the analysis, and TPM values were normalized using the "normalize.quantiles" function in the Bioconductor

preprocessCore package (Bolstad, 2011). After all filtering steps, 205 cells remained from the 228 cells that had productive TCR alignments with 3274 genes.

Single-Cell gene expression comparisons. Out of the 205 cells with reconstructed TCR sequences, 30 were $CD4^+$ and 175 were $CD8^+$ by Flow Cytometry gating. The expression of CD4, CD8A, and CD8B transcripts between the $CD4^+$ and $CD8^+$ cells was compared using Mann-Whitney-Wilcoxon test in R to determine the independence of CD4, CD8A, and CD8B expression in these populations.

TCR α and β chain sequencing

In order to reconstruct CDR3 sequences from single-cell RNA-sequencing data we developed TrapeS ("TCR Reconstruction Algorithm for Paired-End Single cells"), a software package for reconstruction of TCR sequences using short (~25bp) single cell paired-end RNA-sequencing. 150bp single-end reads were converted to artificial 49 bp read pairs for TrapeS analysis. TrapeS first takes standard genomic alignments as input and identifies the genomic segments that constitute the TCR by selecting the V and J segments expressed in the cell. Next, TrapeS takes the unmapped mates of the reads mapped to the genomic segments of the TCR and reconstructs the CDR3 region using an iterative dynamic programming algorithm. In each iteration the reads are aligned to the V and J segments, allowing only partial alignment to the ends of the segments so the reads "flank" toward the CDR3 region (flank the 3' end of the V segment and the 5' end of the J segment). Our method then extends the V and J regions using the sequence of the aligned reads, and repeats this step iteratively until the reconstructed regions overlap. TrapeS is available upon request.

Preparation of soluble DR11-Gag41

As previously described (Crawford et al., 2006; Crawford et al., 1998), soluble DR11-Gag41 was expressed as soluble protein in baculovirus infected insect cells with the Gag41 peptide covalently attached via a C-terminal flexible linker to the N-terminus of the DR11 beta chain. A stabilizing C terminal acid-base leucine zipper was added to the C-terminal end of the DR11 alpha and beta chains and a peptide tag for enzymatic biotinylation was added to the c-terminus of the DR11 alpha chain. After purification this tag was biotinylated enzymatically. To prepare fluorescent tetramers, phycoerythrin-coupled streptavidin (PE-SA) was incubated with an excess of the biotinylated DR11-Gag41 or a control mouse IA^b-p3K protein and the fluorescent complex separated from the excess MHCII by size exclusion chromatography.

Preparation of soluble TRAV6-TRBV2 TCR.

As previously described (Dai et al., 2008; Tynan et al., 2005), the TRAV6-TRBV2 V domains were expressed separately in bacterial vectors fused to human Cα or Cβ. The separate chains were denatured and solubilized from inclusion bodies, mixed and refolded to form a native TCR.

Surface plasmon resonance (SPR)

SPR studies were performed with a BIAcore 2000 instrument containing a SA biosensor chip. ~2000 resonances units (RU) of biotinylated either DR11-Gag41 or control HLA-DR52c bound to a nickel mimicking peptide were captured in separate flow cells. Various concentrations of the soluble TRAV6 and TRBV2 TCR were injected for 80 seconds at 15 uL/min. Binding affinity was calculated from the association and dissociation curves using BIAEvaluation 4.1 software after subtracting the fluid phase RU signal seen with the control DR52c complex.

SUPPLEMENTAL REFERENCES

Bolstad, B.M. (2011). preprocessCore: A collection of pre-processing functions. R package version 1.32.0.

Chen, H., Ndhlovu, Z.M., Liu, D., Porter, L.C., Fang, J.W., Darko, S., Brockman, M.A., Miura, T., Brumme, Z.L., Schneidewind, A., *et al.* (2012). TCR clonotypes modulate the protective effect of HLA class I molecules in HIV-1 infection. Nat Immunol *13*, 691-700.

Crawford, F., Jordan, K.R., Stadinski, B., Wang, Y., Huseby, E., Marrack, P., Slansky, J.E., and Kappler, J.W. (2006). Use of baculovirus MHC/peptide display libraries to characterize T-cell receptor ligands. Immunol Rev *210*, 156-170.

Crawford, F., Kozono, H., White, J., Marrack, P., and Kappler, J. (1998). Detection of antigen-specific T cells with multivalent soluble class II MHC covalent peptide complexes. Immunity *8*, 675-682.

Dai, S., Huseby, E.S., Rubtsova, K., Scott-Browne, J., Crawford, F., Macdonald, W.A., Marrack, P., and Kappler, J.W. (2008). Crossreactive T Cells spotlight the germline rules for alphabeta T cell-receptor interactions with MHC molecules. Immunity *28*, 324-334.

Henn, M.R., Boutwell, C.L., Charlebois, P., Lennon, N.J., Power, K.A., Macalalad, A.R., Berlin, A.M., Malboeuf, C.M., Ryan, E.M., Gnerre, S., *et al.* (2012). Whole genome deep sequencing of HIV-1 reveals the impact of early minor variants upon immune recognition during acute infection. PLoS Pathog *8*, e1002529.

Hermans, I.F., Silk, J.D., Yang, J., Palmowski, M.J., Gileadi, U., McCarthy, C., Salio, M., Ronchese, F., and Cerundolo, V. (2004). The VITAL assay: a versatile fluorometric technique for assessing CTL- and NKT-mediated cytotoxicity against multiple targets in vitro and in vivo. J Immunol Methods *285*, 25-40.

Jones, R.B., Garrison, K.E., Mujib, S., Mihajlovic, V., Aidarus, N., Hunter, D.V., Martin, E., John, V.M., Zhan, W., Faruk, N.F., *et al.* (2012). HERV-K-specific T cells eliminate diverse HIV-1/2 and SIV primary isolates. J Clin Invest *122*, 4473-4489.

Langmead, B., and Salzberg, S.L. (2012). Fast gapped-read alignment with Bowtie 2. Nat Methods *9*, 357-359.

Li, B., and Dewey, C.N. (2011). RSEM: accurate transcript quantification from RNA-Seq data with or without a reference genome. BMC Bioinformatics *12*, 323.

Miura, T., Brockman, M.A., Brumme, C.J., Brumme, Z.L., Carlson, J.M., Pereyra, F., Trocha, A., Addo, M.M., Block, B.L., Rothchild, A.C., *et al.* (2008). Genetic characterization of human immunodeficiency virus type 1 in elite controllers: lack of gross genetic defects or common amino acid changes. J Virol *82*, 8422-8430.

Trapnell, C., Pachter, L., and Salzberg, S.L. (2009). TopHat: discovering splice junctions with RNA-Seq. Bioinformatics *25*, 1105-1111.

Tynan, F.E., Borg, N.A., Miles, J.J., Beddoe, T., El-Hassen, D., Silins, S.L., van Zuylen, W.J., Purcell, A.W., Kjer-Nielsen, L., McCluskey, J., *et al.* (2005). High resolution structures of highly bulged viral epitopes bound to major histocompatibility complex class I. Implications for T-cell receptor engagement and T-cell immunodominance. J Biol Chem *280*, 23900-23909.
